# Programmable Microwaveable Chemistry in the Chemputer

**DOI:** 10.1002/anie.202515869

**Published:** 2025-11-29

**Authors:** Jacopo Zero, Ekaterina Trushina, Niclas Grocholski, Nikita Smirnov, Dean Thomas, Leroy Cronin

**Affiliations:** ^1^ School of Chemistry University of Glasgow University Avenue Glasgow G12 8QQ UK

**Keywords:** Automation, Chemputation, Digital chemistry, Microwave chemistry, Solid‐Phase synthesis

## Abstract

The advancement of laboratory automation relies on both sophisticated control software and modular hardware capable of supporting diverse chemical processes. While microwave‐assisted synthesis offers significant benefits, its integration into automated platforms has been limited by challenges in standardization, scalability, and safety. Herein, we present an automated platform featuring two distinct microwave modules for temperature control, each offering complementary advantages. The first employs a coaxial antenna delivering up to 450 W at 2.45 GHz for flexible applications across various reactor scales. The second consists of a pressurized flow‐cell cavity with in‐situ infrared sensing for real‐time temperature monitoring. To validate the platform, a series of microwave‐assisted reactions were executed under the abstract control of the Chemical Description Language (χDL). Over 680 χDL base steps capture the synthesis of six compounds through O‐alkylations, Suzuki–Miyaura cross‐couplings, ring‐closing metathesis, and solid‐phase peptide synthesis. Reactions ranged from 2 to 20 h, with volumes from 10 to 250 mL, demonstrating the platform's robustness and versatility. This work extends the Chemputer's capabilities to include safe, scalable microwave‐driven synthesis, establishing a foundation for broader adoption of programmable, automated chemistry.

## Graphical abstract

The Chemputer integrates complementary microwave modules under χDL control, enabling fully automated syntheses of O‐alkylation products, Suzuki–Miyaura cross‐couplings, ring‐closing metathesis, and peptide sequences via solid‐phase methods. This modular, programmable platform delivers flexible and scalable microwave‐assisted workflows, broadening access to diverse synthetic methodologies through precise hardware abstraction and standardized digital execution.

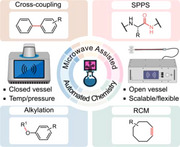

## Introduction

**Figure 1:**
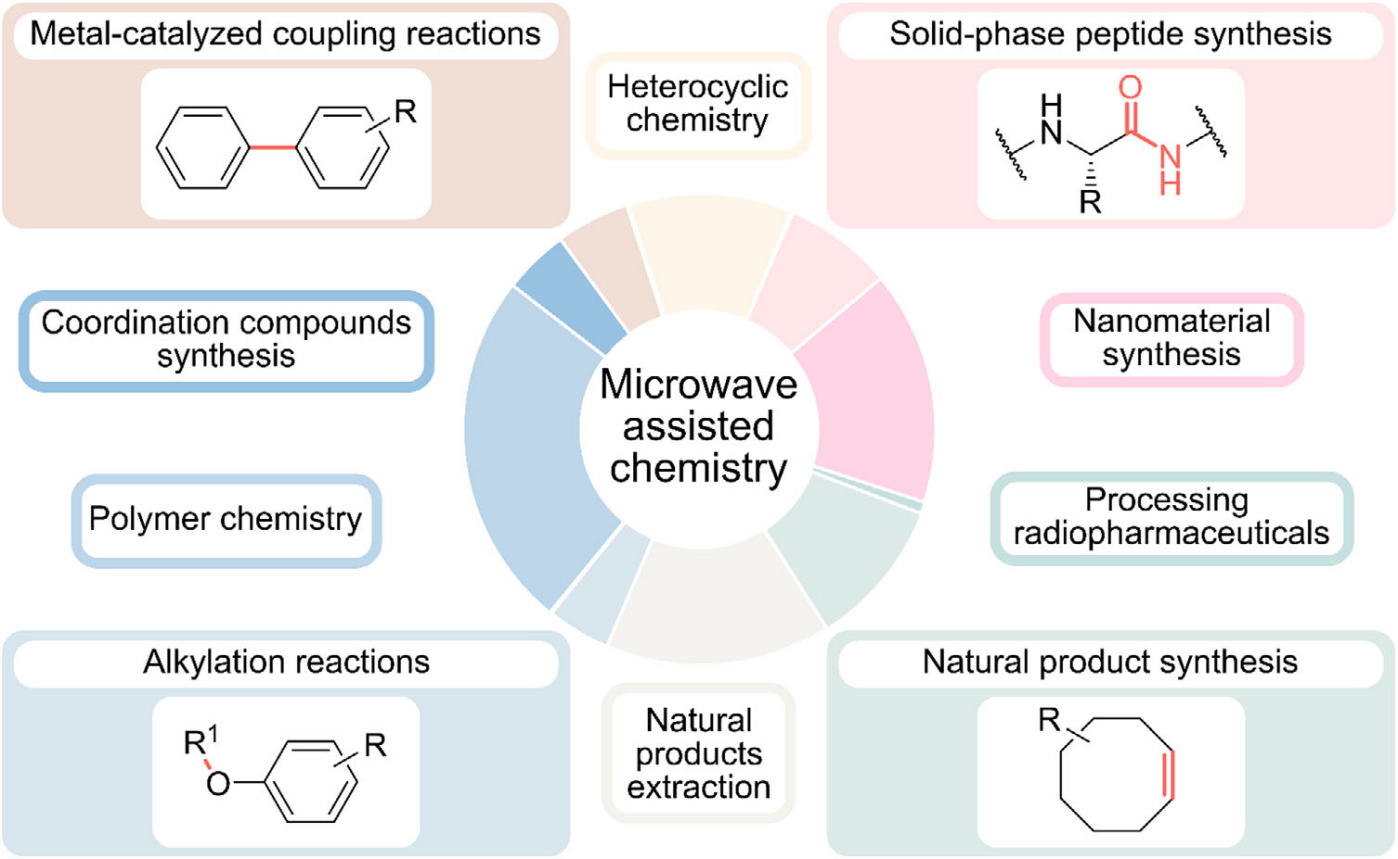
Overview of research fields in microwave‐assisted chemistry. Major research domains in which microwave‐assisted chemistry has been applied, based on a survey of scientific publications. Polymer chemistry 25%, nanomaterial synthesis 16%, natural products extraction 16%, heterocyclic chemistry 11%, natural product synthesis 10%, solid‐phase peptide synthesis 8%, metal‐catalyzed coupling reactions 5%, coordination compounds synthesis 4%, alkylation reactions 4%, processing radiopharmaceuticals 1%.

Since initial reports on the application of microwave irradiation in synthetic chemistry,^[^ ^1^, ^2^ ^]^ microwave‐assisted techniques have evolved from a specialized laboratory procedure into a widely‐adopted methodology for facilitating and accelerating a broad range of chemical transformations. The unique advantages offered, such as rapid and selective heating, enhanced reaction rates, and improved yields, have made microwave irradiation an increasingly attractive tool in both academic and industrial settings.^[^ ^3^ ^]^ Microwave‐assisted synthesis has found applications across numerous domains of chemistry (Figure 1). In radiopharmaceutical production, for instance, microwave‐assisted chemistry enables the rapid labelling of compounds, which is particularly beneficial when time is a critical factor whilst working with short‐lived radioisotopes.^[^ ^4^ ^]^ In materials science, microwave‐assisted methods allow for precise control over the physical and chemical properties of nanomaterials, contributing to advancements in catalysis,^[^ ^5^ ^]^ electronics,^[^ ^6^ ^]^ and biomedical applications.^[^ ^7^ ^]^ Similarly, in coordination chemistry, microwave irradiation is employed to synthesize complex scaffolds such as covalent organic frameworks^[^ ^8^ ^]^ and to develop new structural classes of metal‐based complexes.^[^ ^9^ ^]^ Beyond synthesis, microwave‐assisted techniques have also improved processes like natural product extraction, offering more efficient, environmentally friendly alternatives that require less solvent and time.^[^ ^10^ ^]^ Notably, a substantial body of research has focused on microwave‐assisted organic synthesis (MAOS), highlighting its effectiveness in a wide spectrum of transformations.^[^ ^11^, ^12^, ^13^ ^]^ These include organic reactions such as alkylation^[^ ^14^, ^15^ ^]^ and cross‐coupling,^[^ ^16^ ^]^ the formation of heterocycles,^[^ ^17^ ^]^ and the synthesis of structurally intricate targets like natural products,^[^ ^18^ ^]^ polymers,^[^ ^19^ ^]^ and peptides.^[^ ^20^ ^]^ The continued advancement and optimization of microwave‐assisted methodologies demonstrate their growing importance in modern synthetic chemistry, providing chemists with powerful tools to streamline reactions, improve reproducibility, and reduce environmental impact.^[^ ^3^, ^21^, ^22^ ^]^

**Figure 2:**
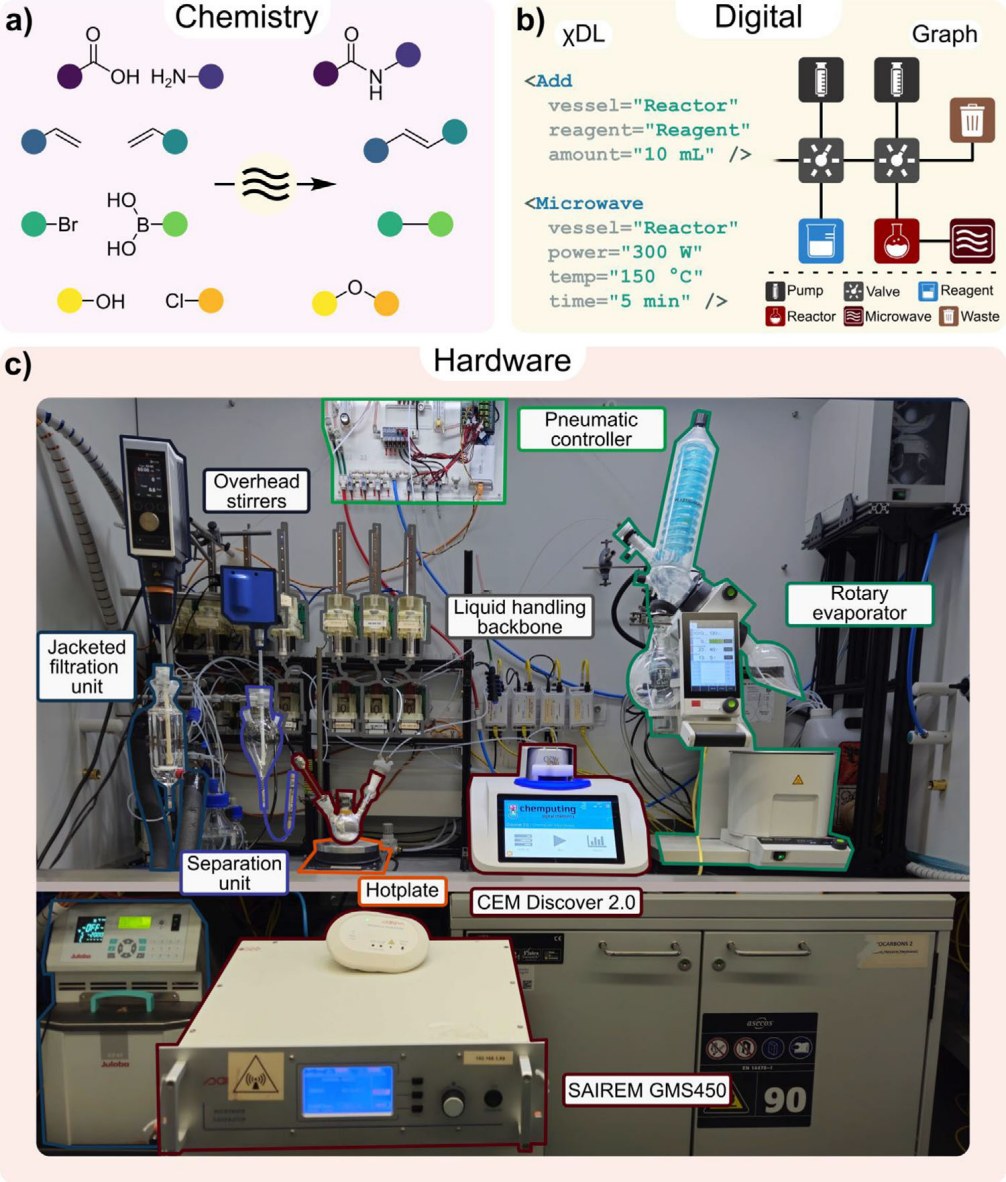
Automated workflow for the microwave‐assisted synthesis on the Chemputer with χDL. a) Microwave‐assisted reactions to automate are selected and synthetic protocols identified. b) Synthetic procedures are translated into χDL digital scripts and the required hardware is represented in a digital graph. c) The physical Chemputer hardware to execute the automated synthesis equipped with the necessary modules, including the two newly introduced microwave generators.

Recent advances in robotics and digital automation are reshaping the landscape of the chemical, biological, and materials sciences, enabling the transformation of laboratory workflows through the automation of both routine and sophisticated tasks.^[^ ^23^ ^]^ These technologies have demonstrated the potential to improve the throughput, safety, and reproducibility of synthetic processes, while also supporting the development of complex molecular architectures.^[^ ^24^, ^25^ ^]^ Nevertheless, the adoption of fully automated synthesis platforms across the broader chemical community has remained limited. This is largely due to the lack of general‐purpose systems capable of accommodating diverse synthetic operations. To overcome these constraints, the Chemical Processing Unit (Chemputer) was developed as a flexible and modular system designed to standardize and automate multi‐step organic syntheses within a unified framework.^[^ ^26^, ^27^, ^28^ ^]^ Operated via the Chemical Description Language (χDL)^[^ ^29^, ^30^ ^]^ the Chemputer platform has demonstrated high chemical flexibility in the synthesis of many different classes of compounds in full automation.^[^ ^27^, ^28^, ^31^, ^32^, ^33^ ^]^ In this study, we build on this foundation to extend the Chemputer's capabilities and address current limitations in synthetic heating strategies by introducing two distinct sources of microwaves for temperature control (Figure 2).

While both microwave systems provide equivalent core functionality, they differ substantially in design and operational parameters, offering complementary advantages depending on the application. One integrated microwave module (SAIREM GMS450) is connected to a coaxial applicator antenna capable of delivering power up to 450 W at 2.45 GHz. It supports scalable reaction volumes as well as custom glassware under ambient pressure with open vessel (OV) conditions, thus enabling flexible adaptation to a broad range of batch‐scale synthetic operations and seamless deployment across various reaction setups. In contrast, the second integrated microwave module (CEM Discover 2.0) incorporates a sealed monomode microwave cavity designed for high‐precision, pressure‐tolerant reactions. It is equipped with an 80 mL fixed‐volume flow cell, an in‐situ infrared (IR) temperature sensor, and a real‐time closed‐loop temperature control system. These features collectively provide stringent control over reaction parameters, taking advantage of all aspects of microwave‐assisted chemical synthesis. To evaluate and validate the performance of these modules within the automated Chemputer framework, a series of representative and synthetically relevant transformations were executed in complete automation across both systems. The selected reactions encompassed a variety of bond‐forming processes and synthetic strategies, including O‐alkylation, C─C bond formation via Suzuki–Miyaura cross‐coupling, ring‐closing metathesis (RCM), and solid‐phase peptide synthesis (SPPS), demonstrating the robustness and adaptability of the platform in agnostically controlling different microwave instruments and executing diverse synthetic protocols with differing operational profiles.

## Results and Discussion

The integration of microwave‐assisted heating into automated chemical platforms presents several design challenges that must be balanced. Most commercially‐available microwave reactors are typically operated as sealed systems, in response to the expected rise in pressure within the flask upon irradiation.^[^ ^34^ ^]^ These closed configurations however limit user and machine accessibility, often making them unsuitable for incorporation into fully automated synthetic workflows, where facile reagent delivery and rapid sampling are essential. Second, the size of these devices must be optimized to ensure compliance with spatial and safety requirements of standard fume hoods, which are commonly used in both academic and industrial laboratories.

To address these limitations, two distinct microwave modules with specific feature sets were incorporated into the Chemputer framework. The first approach utilizes the SAIREM GMS450 microwave generator and a flexible coaxial antenna.^[^ ^35^, ^36^ ^]^ The radiating segment of the antenna is encased in a glass sheath to protect against corrosion or cross‐contamination and inserted directly into a reaction vessel (Figure S7). This method enables minimal spatial occupation within the fume hood while offering direct microwave energy delivery to any reaction flask, independent of a traditional cavity‐based waveguide setup. To ensure operational safety, the reactor was equipped with a brass choke positioned at the antenna's glassware interface to minimize backward microwave propagation. Additionally, reactors were wrapped in aluminum foil/mesh allowing visual inspection whilst preventing radiation leakage. Containment efficiency was verified using a TEK500 contact microwave leakage detector (Martindale Electric Co. Ltd), and continuous monitoring was implemented via a DFM M24DC microwave survey meter (SAIREM), which was interfaced with the system's interlock to automatically disable the generator in the event of leakage detection (further information in Supporting Information 2.1). The use of a coaxial waveguide and antenna reduced the fume hood footprint of the microwave module to that of standard glassware. Furthermore, this approach is inherently modular, allowing for interchangeable vessel shapes and sizes in a ‘plug and play’ fashion, thus increasing experimental flexibility. However, a configurational limitation of such a modular interface is that the device can only operate under ambient pressure, restricting its use in pressurized reactions.

In contrast, the CEM Discover 2.0 monomodal microwave reactor supports reaction temperatures up to 300 °C and closed vessel (CV) conditions with pressures up to 30 bar. The module is equipped with a magnetic stirrer, an infrared temperature sensor and operates through a real‐time closed‐loop temperature control system, ensuring the accurate temperature is maintained throughout the course of a reaction. Crucially, the system incorporates a pressurized 80 mL flow cell, capable of interfacing with external liquid‐handling modules, thus permitting automated reagent addition and sampling via a dedicated inlet and outlet port (Figure S9). This configuration enables the full exploitation of microwave‐assisted synthesis benefits within a fully automated workflow, unlocking new possibilities in chemical automation. Despite its configurational flexibility, the module remains constrained by its reliance on a cavity‐based waveguide, imposing inherent limitations on the geometry and scalability of reaction vessels.

**Figure 3:**
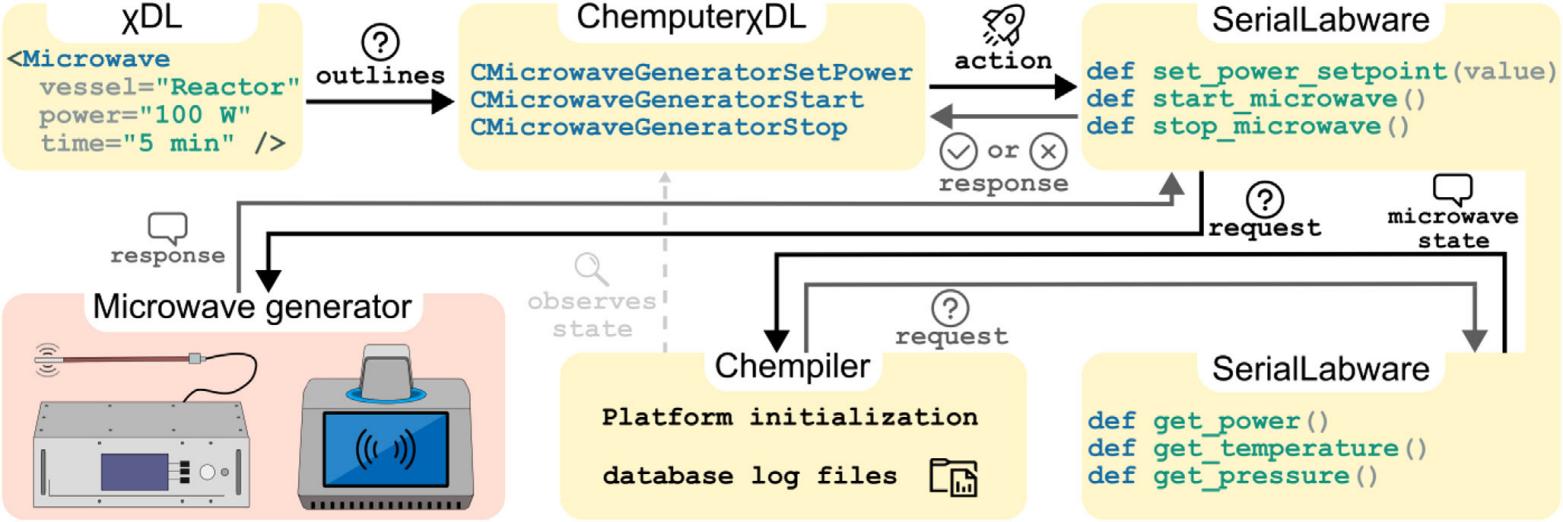
Remote control of microwave devices via χDL. When a Microwave χDL step is executed, a request is sent to the ChemputerχDL library which generates executable, platform‐specific, instructions. Communication with the microwave generator is implemented via the SerialLabware library. The Chempiler is responsible for recording the instrument's activity in digital log files after initialization of the platform. ChemputerχDL will initiate the irradiation protocol and monitor positive or negative responses of the microwave generator in operation.

When integrating new devices into the Chemputer software stack for automated synthesis, it is of critical importance to ensure that bespoke device commands offering remote device control conform to the overarching abstract standard of the Chemical Description Language. This is foundational to the broad (co‐)operability and modularity of χDL. Therefore, following the abstraction principles of the scripting language, a dedicated Microwave step was introduced in the χDL library with the implementation of the new sources of irradiation. This allows the specification of irradiation power, as well as temperature and pressure, depending on the device's capabilities. Two essential software layers interact with the device (Figure 3). Firstly, the platform‐independent, human‐readable χDL code is parsed by the χDL module to be interpreted by the Chemputer's platform‐specific implementation of the scripting language, ChemputerχDL, yielding executable, device‐specific instructions. These instructions are, in a second step, passed to the SerialLabware library, which communicates with the laboratory hardware. Communication from the χDL software layer is accompanied by regular requests through the Chempiler library which records the state of the microwave generator to store data in log files for informational purposes.

**Scheme 1:**
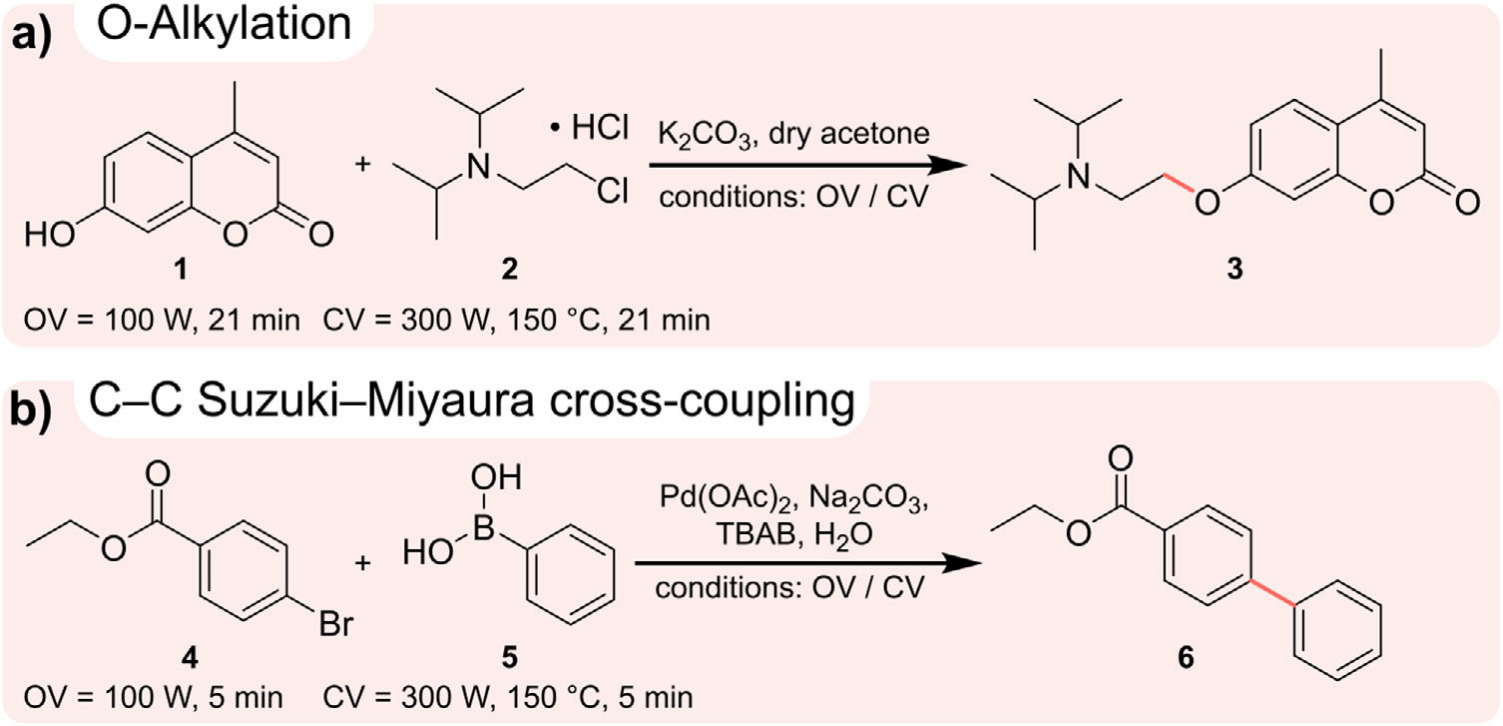
Automated, microwave‐assisted chemical reactions at ambient and pressurized conditions. a) O‐Alkylation of 7‐hydroxy‐4‐methylcoumarin with 2‐diisopropylaminoethyl chloride hydrochloride. b) C─C Cross‐coupling Suzuki–Miyaura reaction of ethyl 4‐bromobenzoate and phenylboronic acid.

To validate the newly introduced microwave modules on the Chemputer platform under χDL control, a series of reactions representative of microwave‐assisted chemical synthesis were selected. Firstly, the O‐alkylation reaction of 7‐hydroxy‐4‐methylcoumarin (**1**) was conducted under ambient pressure with 2‐diisopropylaminoethyl chloride hydrochloride (**2**) in dry acetone in the presence of anhydrous K_2_CO_3_ (Scheme 1a).^[^ ^15^ ^]^ The automated procedure subjected the heterogenous mixture to microwave irradiation (100 W, 3 x 5 min with 2 min wait time between irradiation cycles) at ambient pressure and subsequently filtered the crude material from the excess base with an in‐line filter before precipitating the product in ice‐cold water. The solution was filtered off and the solid dried under vacuum to yield 7‐[2‐(*N*,*N*,‐diisopropylamino)ethoxy]‐4‐methylcoumarin (**3**) (56%).

Subsequently, the automated Suzuki–Miyaura cross‐coupling of ethyl 4‐bromobenzoate (**4**) and phenylboronic acid (**5**) with Pd(OAc)_2_ was executed under ambient pressure (Scheme 1b).^[^ ^37^ ^]^ The mixture was heated under microwave irradiation (100 W, 5 min) then left to cool to room temperature. After diluting with water, the product was extracted with diethyl ether using an on‐line separator equipped with a conductivity sensor. The combined organic layers were then passed through a drying cartridge and the solvent evaporated under reduced pressure in a rotary evaporator. Finally, the product was transferred to an automated flash chromatography system for purification to yield ethyl 4‐phenylbenzoate (**6**) (49%).

The same reactions were then executed in the sealed cavity‐based flow cell in closed vessel conditions, at pressure, and monitored temperature using the CEM Discover 2.0 microwave reactor in automation under χDL control. Due to the agnostic nature of the Chemical Description Language, it was possible to automate these reactions under different conditions and instruments without changes in the χDL digital script using the same Microwave step with updated device specific parameters. Compounds **3** and **6** were then synthesized in automation under pressurized conditions (Scheme 1a,b) following the same protocols highlighted for the open vessel syntheses but different irradiation profiles, giving comparable results (64% and 57% yield respectively).

**Scheme 2:**
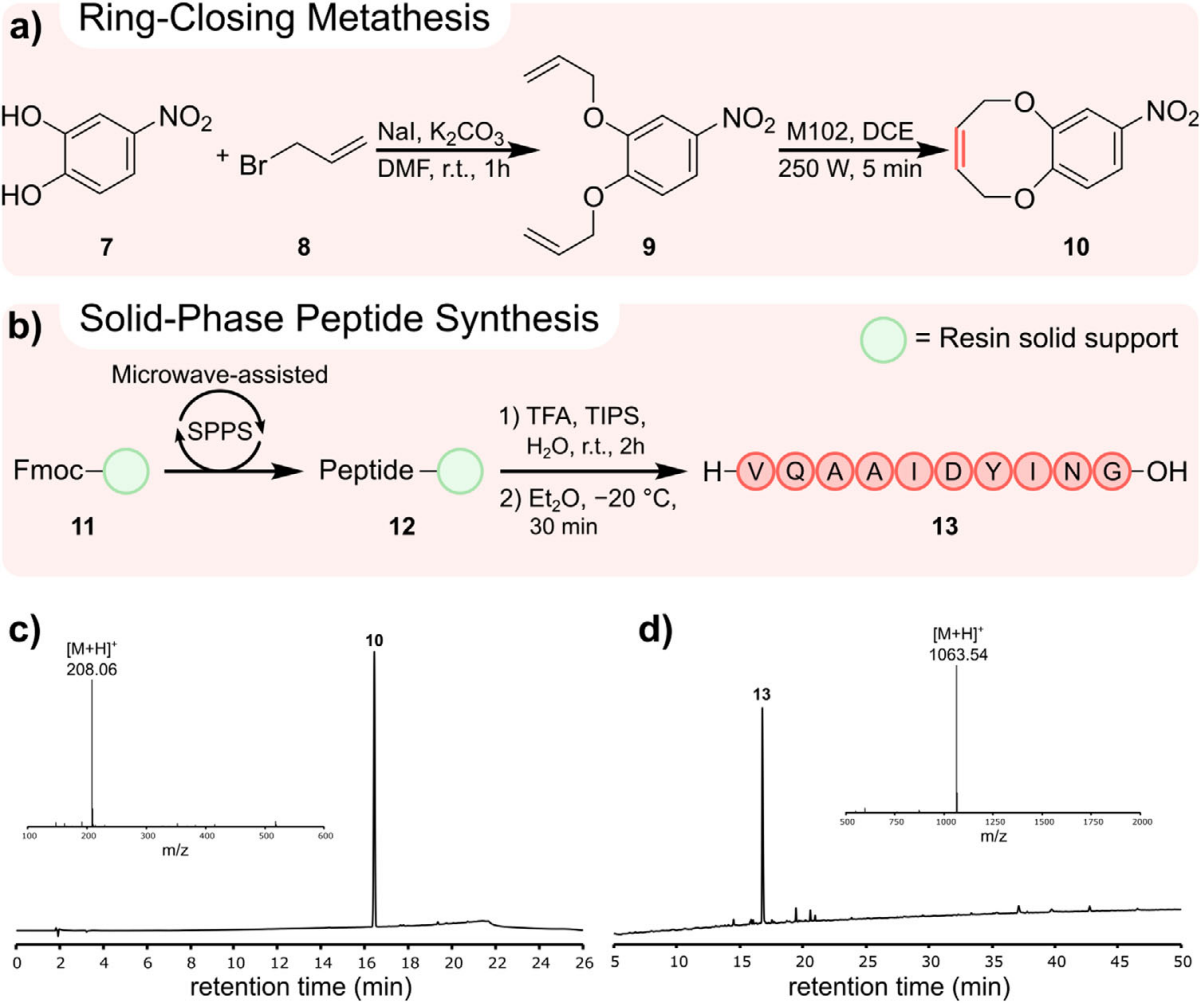
Automated microwave‐assisted chemical reactions with specific process requirements. a) Two‐step microwave‐assisted ring‐closing metathesis. b) Microwave‐assisted solid‐phase peptide synthesis of ACP(65‐74). c) RP‐HPLC trace (254 nm, 19 min 0%–80% MeCN gradient) and ESI‐MS spectra (inlet) of **10**. d) RP‐HPLC trace (280 nm, 60 min 0%–80% MeCN gradient) and ESI‐MS spectra (inlet) of **13**.

Following O‐alkylation and cross‐coupling, two synthetic procedures with strict process control requirements were then automated with microwave irradiation to demonstrate the advantages of the Chemputer's modular nature. The first of which being a microwave‐assisted ring‐closing metathesis to yield the eight‐membered ring (*Z*)‐8‐nitro‐2,5‐dihydro‐benzo[b][1,4]dioxocane (**10**), common structural element of many natural products (Scheme 2a).^[^ ^38^ ^]^ In order to carry out this reaction, the Chemputer system, including the rotary evaporator, was kept under an argon atmosphere fed through the pneumatic controller. The preparative phase of the automated synthesis involved χDL‐controlled vacuum and inert gas cycling of the M102 Grubb's first‐generation ruthenium catalyst vial. Following preparation, the first step involved the automated synthesis of 1,2‐bis‐allyloxy‐4‐nitro‐benzene (**9**) from 4‐nitrocatechol (**7**) with allyl bromide (**8**) (Supporting Information 4.5).^[^ ^39^ ^]^ The second step of the synthesis involved dissolution of **9** in dry 1,2‐dichloroethane and transfer from the rotary evaporator flask into the 500 mL microwave‐shielded reactor flask. Here, the mixture was purged with argon and a solution of M102 catalyst in dry 1,2‐dichloroethane added, reaching a final volume of 250 mL with a maximum diene concentration of 4 mM. The reaction mixture was then irradiated (250 W, 5 x 1 min with 20 s wait time between irradiation cycles) with argon sparging. After completion of the microwave cycle, the mixture cooled to room temperature before evaporation under reduced pressure in the rotary evaporator. The crude product was then transferred to an automated flash chromatography system for purification to afford compound **10** (42%). RCM reactions inherently benefit from the SAIREM's ambient pressure and scalable approach. Volatile byproducts such as ethylene can escape and drive the catalytic cycle to completion whilst high dilution conditions, only obtainable in large vessels fitted with the coaxial antenna, limit the formation of acyclic diene metathesis side products, verified via reversed‐phase high‐performance liquid chromatography (RP‐HPLC) and electrospray ionization mass spectrometry (ESI‐MS) (Scheme 2c).

The final microwave‐assisted synthesis was the solid‐phase peptide synthesis (SPPS) of the sequence fragment of acyl carrier protein ACP(65‐74) (**13**), commonly used to assess the efficiency of SPPS platforms (Scheme 2b).^[^ ^40^ ^]^ Solid‐phase synthesis requires fritted‐glass reactors of varying sizes and benefit from temperature‐controlled systems, so that peptide elongation cycles of deprotection and coupling can be optimized and then scaled appropriately. Consequently, for this automated process, the SAIREM's flexible microwave coaxial antenna elegantly interfaced with a shielded, fritted‐glass reactor filter (Figure S8). The resin solid support, Fmoc‐Gly‐Wang (**11**), was subjected to cycles of Fmoc deprotection and amino acid coupling to afford the final peptide using a recently developed Chemputer protocol^[^ ^33^ ^]^ adapted for microwave‐assisted heating. Amide couplings consisting of 2 mL Fmoc protected amino acid (0.5 M in DMF), 2 mL coupling reagent hexafluorophosphate azabenzotriazole tetramethyl uronium (HATU) (0.5 M in DMF), and 0.5 mL *N*,*N*‐diisopropylethylamine (DIPEA) were executed at elevated temperature under microwave irradiation (150 W, 5 x 15 s with 45 s wait time between irradiation cycles). Fmoc deprotection consisted of treatment with 9 mL 20% piperidine in DMF under microwave irradiation (150 W, 3 x 15 s with 45 s wait time between irradiation cycles). The resin‐bound, side chain protected peptide fragment **12** was then subjected to cleavage from the solid support using 10 mL cleavage mixture of TFA/H_2_O/TIPS (90:5:5, v/v/v), prepared in automation and sparged with nitrogen for 2 h. The TFA mixture was then transferred into a jacketed filter containing pre‐chilled diethyl ether (Et_2_O) (−20 °C). The mixture was then sparged with nitrogen for 30 min before filtration. Finally, the peptide product was washed with cold Et_2_O, dried under vacuum, dissolved in MeCN/H_2_O (1:1, v/v), and transferred to a receiving flask for collection. After manual lyophilization, compound **13** was obtained in 32% yield and 76% crude purity (Scheme 2d). All stages of SPPS were captured in distinct χDL blueprints which summarize long and repetitive procedures into single χDL steps (Supporting Information 3.3).

## Conclusion

We have demonstrated the successful integration and validation of two microwave modules on the Chemputer platform, operating under χDL control. A representative set of microwave‐assisted reactions were executed to evaluate performance across diverse synthetic conditions. These included O‐alkylation and Suzuki–Miyaura cross‐coupling, both executed under ambient and pressurized conditions highlighting the hardware‐agnostic and procedural flexibility afforded by χDL. To further demonstrate the advantages of the Chemputer's modular design, we implemented synthetic workflows with specific process control requirements under microwave irradiation. These comprised i) a ring‐closing metathesis to form an eight‐membered ring—a core motif in many natural products—conducted under high dilution conditions, and ii) the solid‐phase peptide synthesis of the ACP(65‐74) peptide fragment, a standard benchmark for assessing SPPS performance, using a fritted‐glass reactor filter. Together, these studies expand the Chemputer's synthetic capabilities to include microwave‐assisted transformations under varied conditions. The combination of modular hardware and the flexible abstraction enabled by χDL establishes a robust foundation for the automated execution of a wider range of synthetic methodologies, contributing to the broader goals of digital chemistry and laboratory automation.

## Author Contributions

LC conceived the idea of advancing thermally/radiatively‐assisted technologies in the Chemputer. JZ and ET designed the hardware and conducted the synthesis in automation. Software development was completed by NG and NS. ET, DT, and LC coordinated the research project. The manuscript and supporting information were written by JZ, ET, NG, DT and LC.

## Conflict of Interests

The authors declare no conflict of interest.

## Supporting information



Supporting Information

Supporting Information

## Data Availability

The data that support the findings of this study are available in the Supporting Information of this article.
