# Supplementary material for: Programmable Microwaveable Chemistry in the Chemputer
Source: Angew Chem Int Ed Engl. 2025 Nov 29;65(4):e15869. doi: 10.1002/anie.202515869 (PMC12828470; doi:10.1002/anie.202515869)
Supplement: Supplementary file 1 — Supporting Information [file ANIE-65-e15869-s001.pdf]

Supporting Information

Programmable Microwaveable Chemistry in the Chemputer

Jacopo Zero[a], Ekaterina Trushina[a], Niclas Grocholski[a], Nikita Smirnov[a], Dean Thomas[a],

Leroy Cronin*[a]

[a] - *School of Chemistry, University of Glasgow, University Avenue, Glasgow G12 8QQ, UK.*

Email: lee.cronin@glasgow.ac.uk

Contents

1. General remarks 3

2. Chemputer hardware 6

2.1 SAIREM GMS450 8

2.2 CEM Discover 2.0 15

3. Chemputer and χDL software 16

3.1 Hardware Integration into the Software Stack 16

3.2 Execution of χDL Files for Automated Syntheses 19

3.3 χDL blueprints for automated microwave-assisted SPPS 20

4. Fully automated microwave-assisted chemical synthesis 23

4.1 Ambient pressure open vessel microwave-assisted O-alkylation (3) 24

4.2 Pressurized closed vessel microwave-assisted O-alkylation (3) 27

4.3 Ambient pressure open vessel microwave-assisted C–C Suzuki–Miyaura cross-coupling (6) 30

4.4 Pressurized closed vessel microwave-assisted C–C Suzuki–Miyaura cross-coupling (6) 34

4.5 Ambient pressure open vessel microwave-assisted ring-closing metathesis (RCM) (10) 37

4.6 Ambient pressure open vessel microwave-assisted SPPS (13) 43

5. References 44

1. General remarks

Solvents and reagents were obtained dried and distilled from commercial sources and used as received unless stated otherwise.

NMR measurements were performed on a Bruker Avance III HD 600 spectrometer operating at 600.1 and 150.9 MHz for ^{1}H and ^{13}C, respectively. Spectra were collected at 298 K, chemical shifts are reported in ppm and were calibrated for the (residual) NMR solvent signal (multiplicities are given as s: singlet, d: doublet, t: triplet, q: quartet, hept: heptet, m: multiplet, with coupling constants reported in Hz). The spectra were processed with MestReNova 15.1.0.

RP-HPLC ESI-MS analysis was performed on a Thermo Dionex Ultimate 3000 HPLC system equipped with an LPG-3400 RS pump, WPS-3000TRS autosampler, TCC-3000SD column compartment, and DAD3000 diode array detector. The HPLC was connected to a Bruker Maxis Impact II HDMS Q-TOF spectrometer (Bruker Daltonics). Samples were injected for chromatographic separation on an Agilent Poroshell 120 EC-C18 column (2.7 µm, 4.6 x 150 mm), eluting at 1 mL min^{-1} with mobile phase A being H_2O + 0.1% formic acid and mobile phase B MeCN + 0.1% formic acid, detecting UV (λ = 214, 220, 254, and 280 nm). The total run time was 73 minutes, with the LC method as follows: 0 min – 1% B, 60 min – 80% B, 62 min – 100% B, 66 min – 100% B, 68 min – 1% B, 73 min – 1% B. Alternatively, the total run time was 26 minutes, with the LC method as follows: 0 min – 0% B, 4 min – 10% B, 16 min – 70% B, 19 min – 100% B, 23 min – 0% B, 26 min – 0% B. Column compartment was set at 30 °C. MS measurements were taken in positive electron spray ionization mode (ESI+) with a mass range of m/z 50–2000 using the following parameters: capillary tip 4500 V, end plate offset -500 V, nebulizer 2.0 bar, dry gas 10.0 L min^{-1}, dry temperature 200 °C, quadrupole ion energy 5 eV, and collision energy 5 eV. Peptide purity was assessed by RP-HPLC at 280 nm using the BrukerAnalysis v4.1 software suite. Yields were calculated based on resin loading, adjusting for TFA counterions at free N-termini as well as purity values obtained from HPLC-MS.

Standardized Operating Procedures were developed and implemented to ensure consistently safe operation conditions. To prevent issues such as clogging or unwanted precipitation within the liquid handling backbone, cleaning protocols were integrated throughout all automated synthetic procedures and in-line filters used where adequate. In the event of hardware disconnection or malfunction, the system automatically halts operation and generates log files documenting the issue. To prevent inefficient energy transfer and potential overheating of the coaxial cable under continuous, high-power microwave operation under remote device control, power was applied in controlled irradiation cycles. Additionally, each

commercial device retains its built-in ‘watchdog’ safety mechanisms, which remain active during remote operation, ensuring correct and safe instrument use at all times. Finally, as a fundamental level of user protection, a Wall-Mount Microwave Surveymeter DFM M24DC 2450 MHz with sounding alarm was fitted and used in conjunction with a Martindale TEK500 detector to identify any leakage of microwave irradiation, as per the manufacturer's instructions.

Supplementary Data contains all digital files required for reproducing the automated syntheses reported in this work. Each folder corresponds to a specific compound and includes the following files:

1. **χDL (.xdl):** The digital procedure file describing the stepwise chemical synthesis.
2. **Graph (.json):** The digital graph file representing the Chemputer hardware.
3. **Compiled χDL (.xdlexe):** The executable version of the χDL file.
4. **Blueprints (.xdl):** The χDL blueprint files used to encompass long procedures into a single χDL step.
5. **Script execution notebook (.ipynb):** The Jupyter notebook used to run the procedure.

Files are organized and numbered according to the compound numbering used in the main manuscript. Folder descriptions are as follows:

Compound 3:
Contains χDL, graph, compiled χDL, and script execution notebook files for:

1. *O-Alkylation_3-1*: Ambient pressure open vessel microwave-assisted O-alkylation (**Supporting Information 4.1**).
2. *O-Alkylation_3-2*: Pressurized closed vessel microwave-assisted O-alkylation (**Supporting Information 4.2**).

Compound 6:
Contains χDL, graph, compiled χDL, and script execution notebook files for:

1. *Cross-coupling_6-1*: Ambient pressure open vessel microwave-assisted C–C Suzuki–Miyaura cross-coupling (**Supporting Information 4.3**).
2. *Cross-coupling_6-2*: Pressurized closed vessel microwave-assisted C–C Suzuki–Miyaura cross-coupling (**Supporting Information 4.4**).

Compound 10:
Contains χDL, graph, compiled χDL, and script execution notebook files for:

1. *RCM_10*: Ambient pressure open vessel microwave-assisted ring-closing metathesis (RCM) (**Supporting Information 4.5**).

Compound 13:

Contains χDL, graph, compiled χDL, blueprints, and script execution notebook files for:

1. *SPPS_13*: Ambient pressure open vessel microwave-assisted SPPS (**Supporting Information 4.6**).

2. Chemputer hardware

The Chemputer hardware utilized in this work is outlined in **Figure S1** and summarized in **Table S1**. All components, excluding the newly introduced microwave modules, were assembled and used as previously described in extensive detail.[1] Detailed construction of the new microwave modules is reported in **Supporting Information 2.1** and **Supporting Information 2.2**.

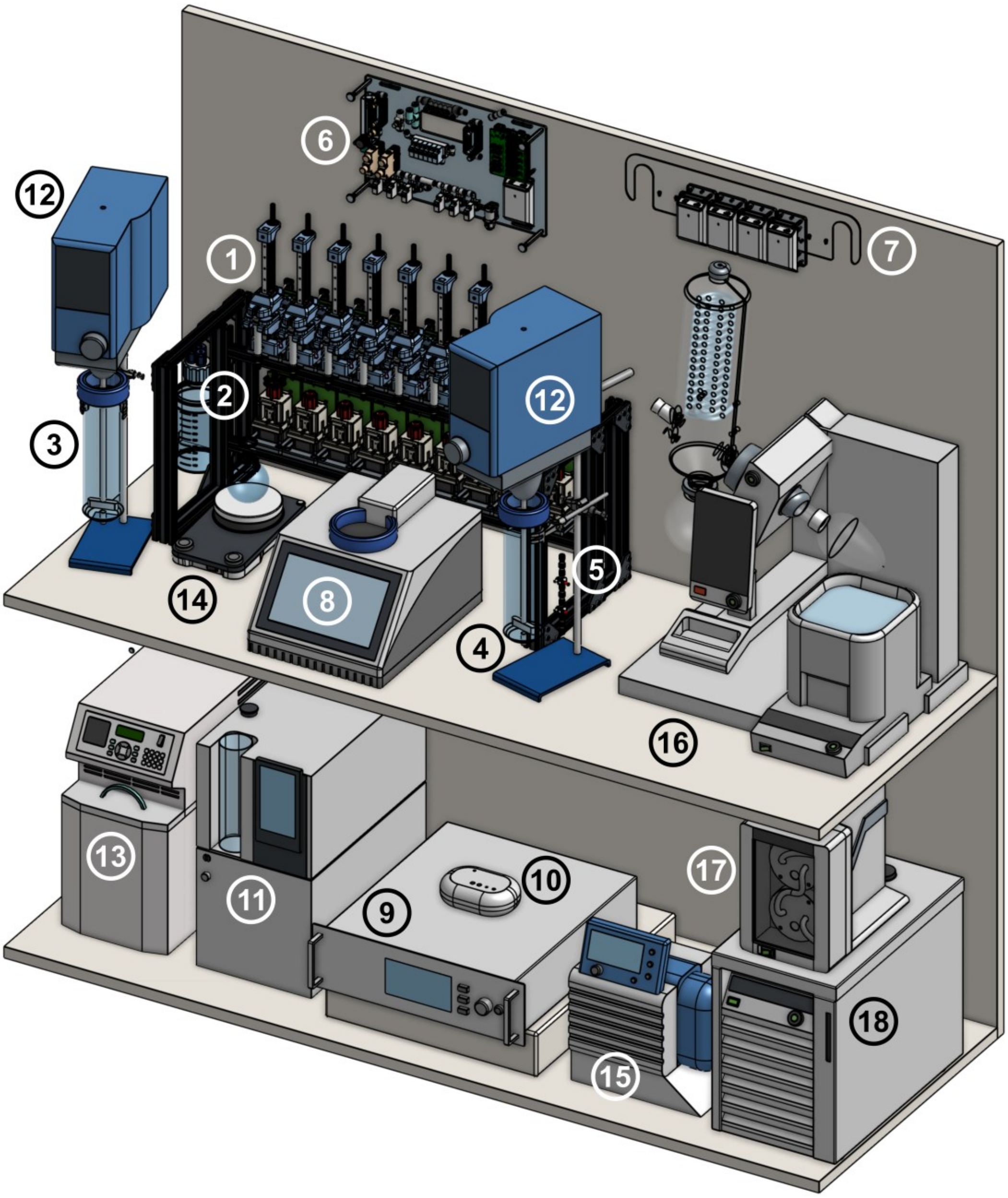

Figure S1. Model blueprint for the microwave Chemputer platform assembly. Hardware components are detailed in Table S1. PTFE tubing used to connect modules to the liquid handling backbone and Viton tubing used to connect the chillers to the appropriate device not shown.

Table S1. List of all hardware components required for the microwave Chemputer platform assembly.

#	Device	Amount	Supplier
1	Chemputer pump	7	In house
2	Chemputer valve	9	In house
3	Chemputer jacketed filter	1	In house – glassware
4	Chemputer separator	1	In house – glassware
5	Conductivity sensor	1	In house
6	Pneumatic controller	1	In house
7	Serial-to-Ethernet converter	4	In house
8	CEM Discover 2.0	1	CEM
9	SAIREM GMS450	1	SAIREM
10	Wall-mount 2450 MHz Microwave Surveymeter	1	SAIREM
11	Huber Petite Fleur chiller	1	Huber
12	Heidolph Hei-TORQUE 100 Precision Base overhead stirrer	1	Heidolph
13	Julabo CF41 chiller	1	Julabo
14	IKA RCT digital hotplate	1	IKA
15	Vacuubrand MD1C vario PLUS with CVC 3000 controller vacuum pump	1	Vacuubrand
16	Buchi R-300 rotary evaporator	1	Buchi
17	Buchi V-300 vacuum pump	1	Buchi
18	Buchi F-305 chiller	1	Buchi
19	PTFE tubing 3.2 mm OD x 1.5 mm ID	varying length	Diba
20	Viton tubing 10 mm OD x 8 mm ID	varying length	Julabo

2.1 SAIREM GMS450

The commercially available 2.4-2.5 GHz 450W solid state microwave generator, SAIREM GMS450, was fully integrated with the liquid handling and logical control of the Chemputer and χDL. The required components for its full assembly are listed in **Table S2** below along with the steps required.

Table S2. Bill of materials for the full assembly of the coaxial antenna SAIREM GMS450 solid state microwave generator module.

#	Description	Manufacturer's part number	Amount	Supplier
**H1**	2.45 GHz 450W solid state microwave generator	GMS450WSM	1	SAIREM
**H2**	Flexible RF cable, 50 Ohm, 18 GHz, 85°C, D 5.3 mm, PUR jacket, 7/16 type male connector, "N" male connector	S_04212_B, 11_716-50-4-5/033_-Y, 21_N-50-4-6/133_NH	1500 mm	HUBER+SUHNER
**H3**	Semi-Rigid Microwave Cable, RG401 dimension, MIL style, 50 Ohm, 18 GHz, 100°C, D 6.35 mm, no jacket, "N" female connector	SR_250_M17, 11_N-50-5-18/103_N	150 mm	HUBER+SUHNER
**H4**	Glass sheath	N/A	1	In house – glassware
**H5**	Choke section, brass H62, bead blasted surface finish	N/A	1	In house – design, Geomiq – manufacturing
**H6**	M3 x 10mm Full Threaded Low Cap Screws (DIN 7984) - Marine Stainless Steel (A4)	SSCL-M3-10-A4	2	AccuGroup

**H7**	M3 Hexagon Nuts (DIN 934) - A4 Stainless Steel	HPN-M3-A4	2	AccuGroup
**H8**	Glassware with standard ground joints NS14/23, round bottom or else, various volumes as required	N/A	1	N/A
**H9**	Dimroth condensers with standard ground joints 14/23, 14/23, 160 mm	201-3331	1	VWR
**H10**	Petite Fleur. Dynamic temperature control system / circulation thermostat	1030.0001.01	1	Peter Huber Kältemaschinenbau AG, Germany
**H11**	PVC Flexible Tubing, Transparent, 12mm External Diameter, Reinforced, 40mm Bend Radius	368-0182	varies	RS Components
**H12**	Wall-mount 2450 MHz Microwave Surveymeter	DFM M24DC 2450 MHz	1	SAIREM
**H13**	Fine Aluminium Mod Mesh Wire 50cm x 3m	729270489895	1	Amazon (QG ltd)
**H14**	Hi-Bond HB 710 Conductive Metallic Tape, 25mm x 20m	832-6341	1	RS Components
**H15**	Martindale TEK500 Microwave Leakage Detector, 2450MHz	252-324	1	RS Components
**H16**	Ethernet cable, 5 m	83607	1	Insight

1. The microwave generator **H1** was installed according to the manufacturer’s manuals.

2. The Petite Fleur dynamic temperature control system **H10** was connected to the microwave generator's cooling system with PVC Flexible Tubing **H11** and was filled with water.

3. The wall-mount 2450 MHz Microwave Surveymeter **H12** was installed and connected to the microwave generator according to manufacturer's manual.

4. The microwave generator **H1** was connected to the control switch with an Ethernet cable **H16** making use of the inbuilt Ethernet socket.

5. A semi-rigid coaxial cable **H3** was used as the microwave applicator. The radiating section was obtained by stripping the outer copper conductor layer by 2.5 cm, calculated according to λ/4 (where λ is the radiation wavelength in vacuum) (**Figure S2**).

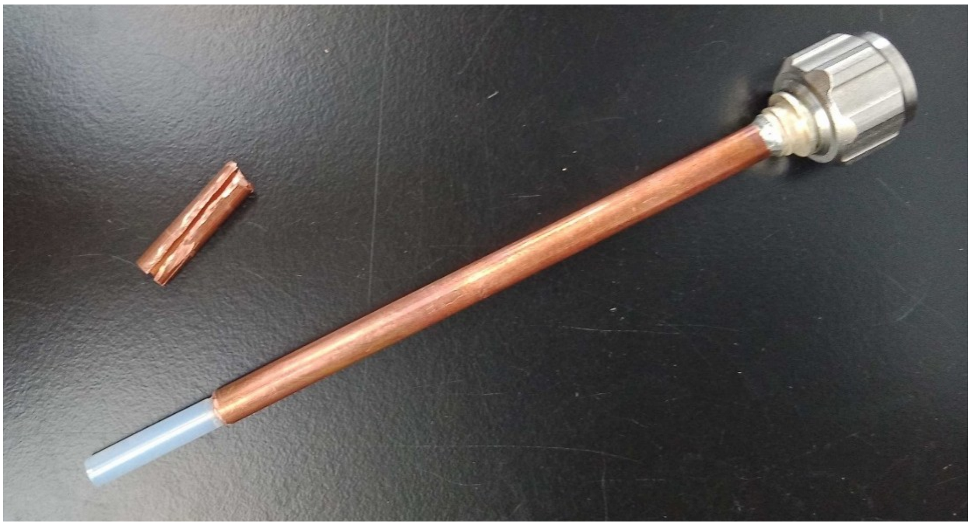

Figure S2. Stripped semi-rigid coaxial cable **H3** used as the microwave applicator.

6. The prepared semi-rigid coaxial cable **H3** was connected to the flexible RF cable **H2** which, in turn, was connected directly to the microwave generator **H1** through the appropriate port according to the manufacturer's manual.

7. Glassware flasks **H8**, three necks round bottom used as an example below, were equipped with aluminum protective coverage prior to experiments. Aluminum coverage was constructed from fine aluminum mesh **H13** and aluminum tape **H14**, aluminium foil was then used to ensure complete coverage of the flask's surface during microwave irradiation experiments (**Figure S3**).

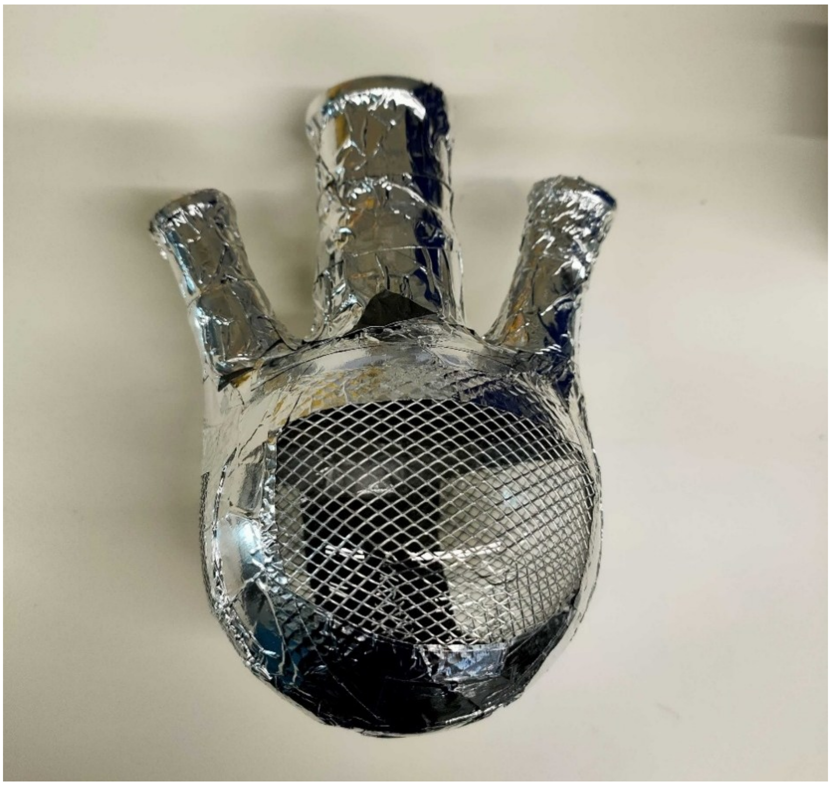

Figure S3. Aluminum covered glassware flask used as microwave reactor.

8. The semi-rigid coaxial cable applicator **H3** was inserted into the brass choke section **H5** (**Figure S4**), covered with glass sheath **H4** (**Figure S5**), and placed in the aluminum shielded flask through the NS14/23 neck. Then, the choke section **H5** was secured with M3 low cap screws **H6** and M3 hexagon nuts **H7** (**Figure S6**).

9. To ensure correct assembly of the microwave reactor flask and the absence of microwaves leaks during irradiation protocols, the setup was monitored using a Martindale TEK500 Microwave Leakage Detector **H15**.

a)

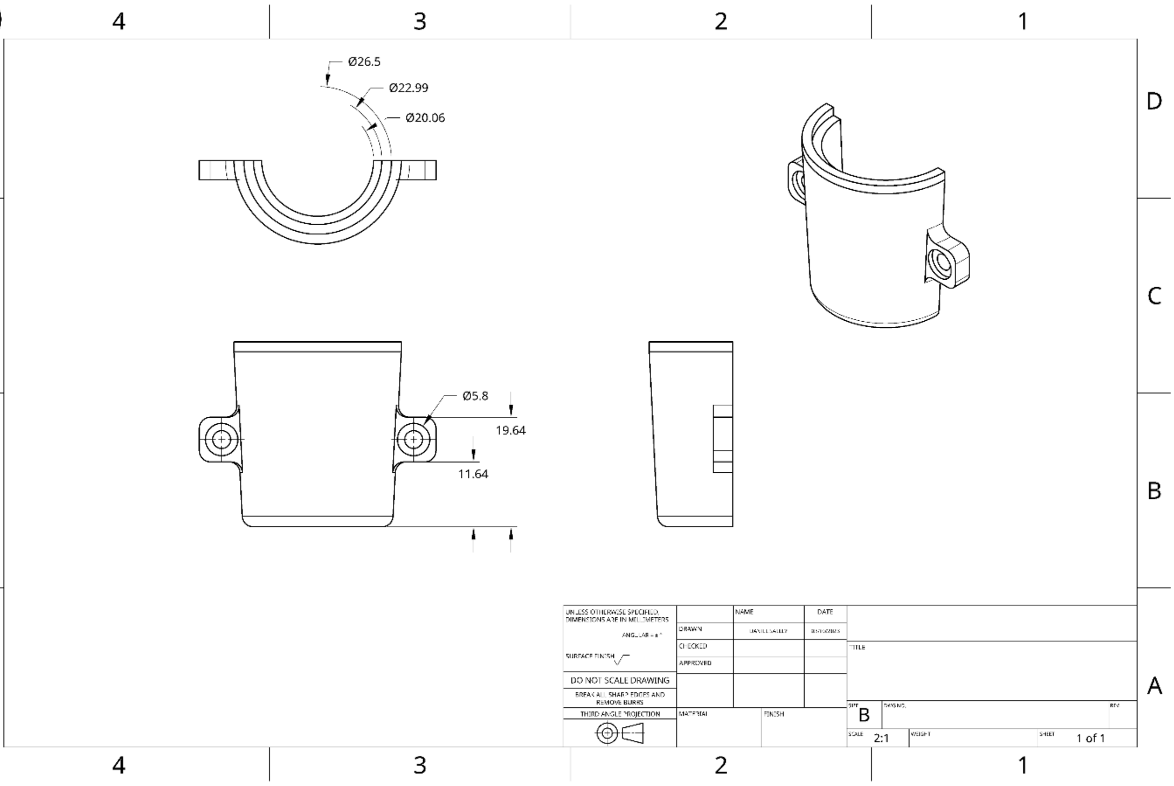

b)

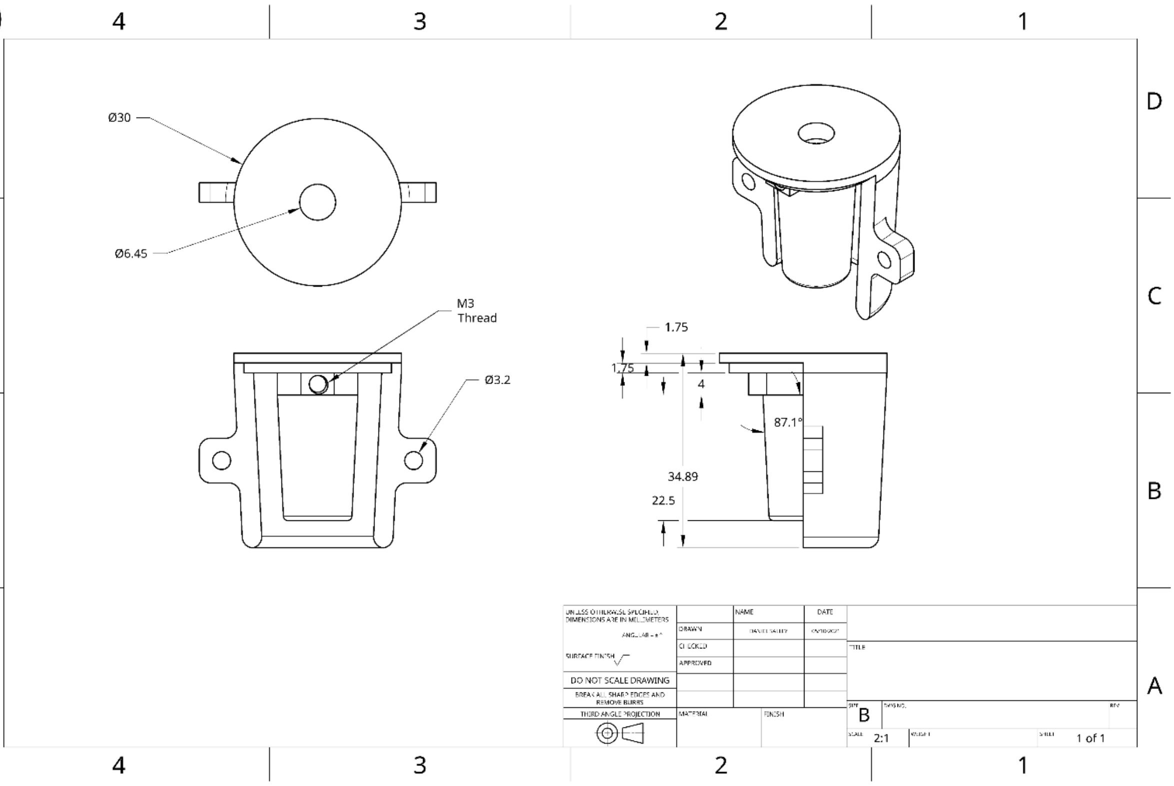

Figure S4. a) Brass choker inner assembly drawing. b) Brass choker outer assembly drawing.

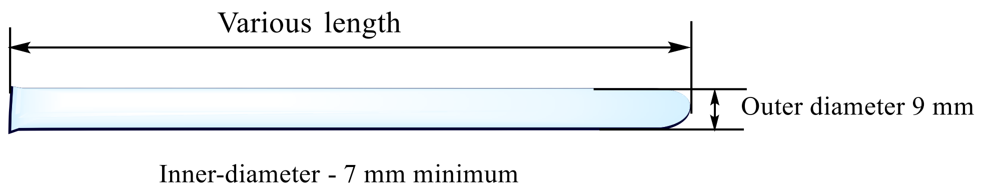

Figure S5. Glass sheath cover scheme for microwave applicator.

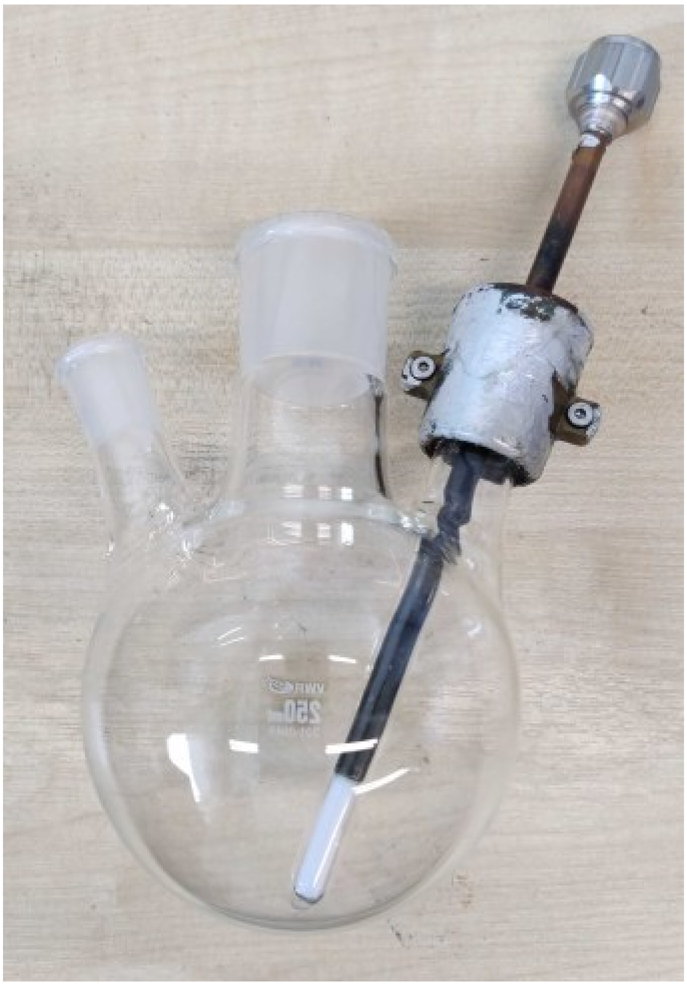

Figure S6. Representation of the microwave reactor setup including the applicator, brass choke, glass sheath, and flask.

10. For reactions requiring reflux conditions, a water-cooled Dimroth condenser **H9** was equipped with the aluminum coverage and applied to the microwave reactor flask as required.

11. The fully assembled microwave module was then directly connected to the liquid handling Chemputer backbone using PTFE tubing as outlined in **Figure S7**. All connection adapters were additionally covered with aluminum tape to ensure safety of the system.

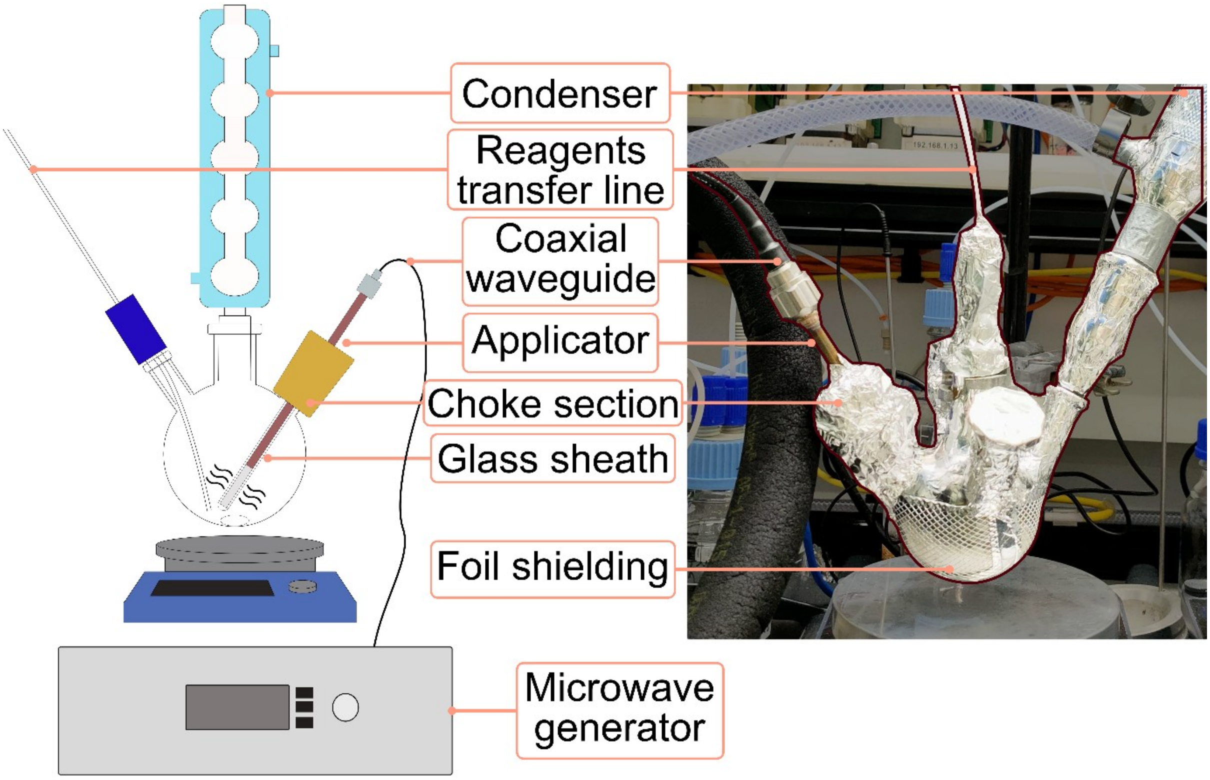

Figure S7. Representative full assembly for the microwave module including the commercial device, coaxial cable, applicator antenna, shielded reactor as required, and PTFE tubing connection to the Chemputer liquid handling backbone.

For the automated execution of microwave-assisted solid-phase peptide synthesis (SPPS), a custom fritted-glass reactor filter was employed (**Figure S8**).

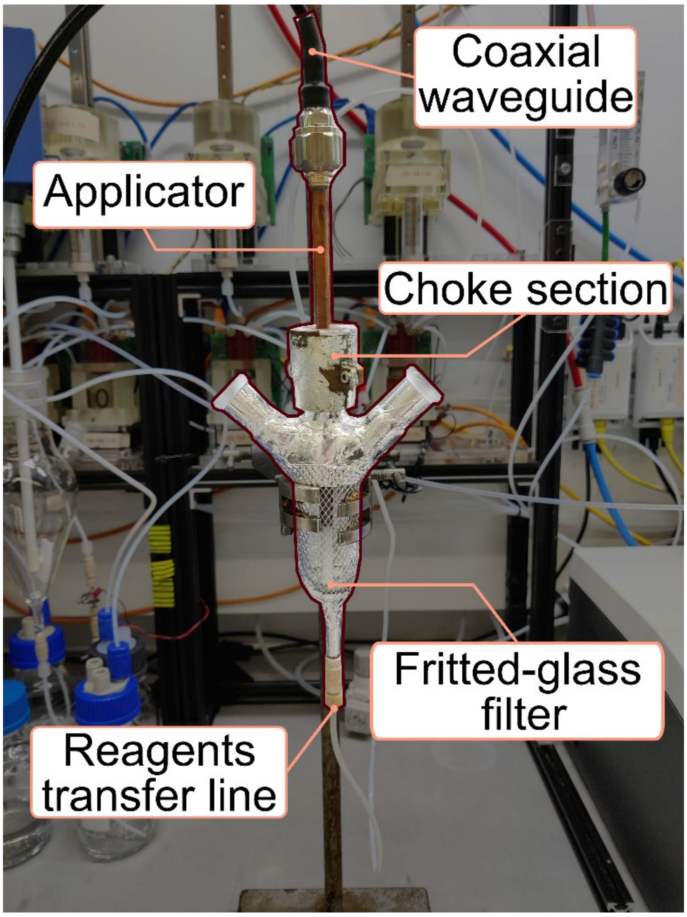

Figure S8. Fritted-glass reactor filter for automated microwave-assisted SPPS on the Chemputer. The filter is equipped with the microwave applicator antenna, fed through the brass choke section, a fritted-glass filter with porosity P4, and a reagents transfer line connecting the Chemputer backbone.

2.2 CEM Discover 2.0

The commercial microwave generator CEM Discover 2.0 was assembled following manufacturer's instructions. The device was operated using the provided 80 mL flow cell which was installed according to the manual (**Figure S9**). The reagents transfer line to and from the flow cell was then connected to the Chemputer's backbone in order to add and remove solutions.

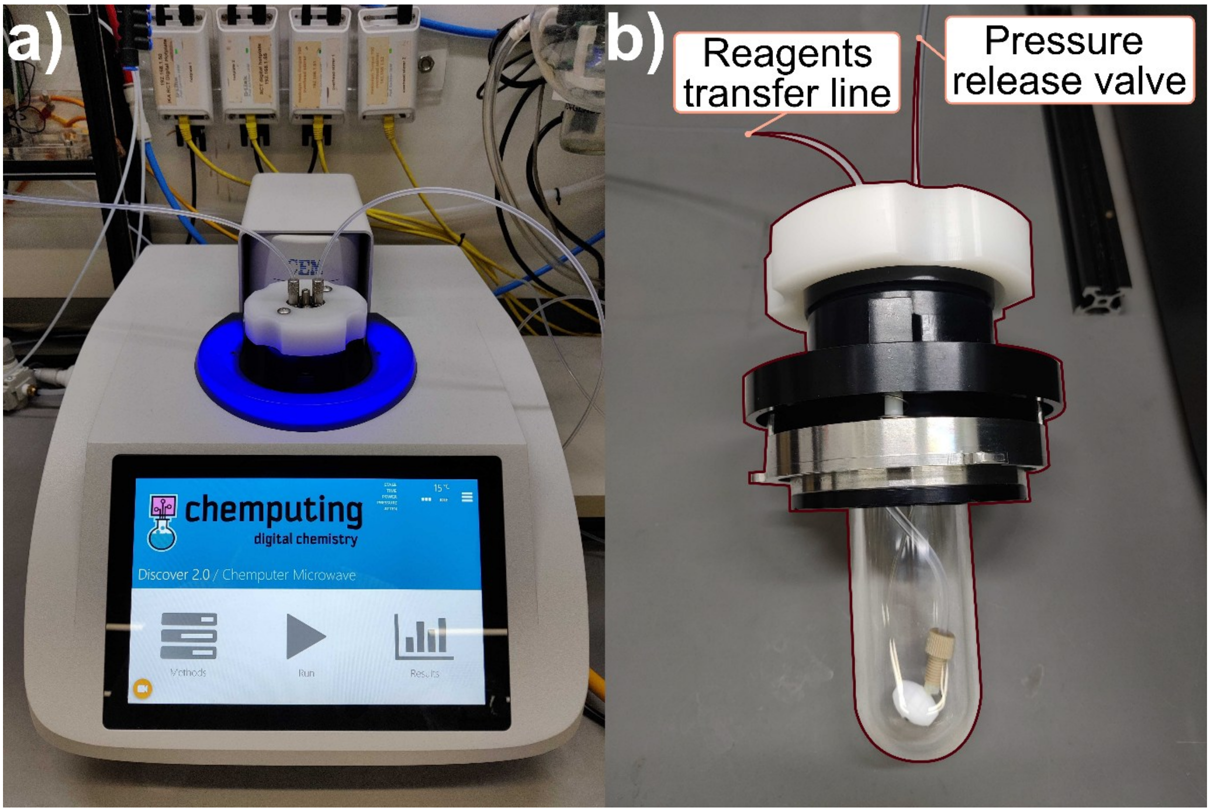

Figure S9. a) CEM Discover 2.0 microwave generator operated via χDL on the Chemputer. The device was assembled following manufacturer’s instructions. b) Provided 80 mL flow cell used to carry out automated microwave-assisted reactions. The flow cell was placed in the microwave cavity and connected to the Chemputer’s backbone via the provided PTFE tubing. The exhaust of the reactor was connected to the provided pressure release valve as detailed in the manufacturer's manual.

3. Chemputer and χDL software

The χDL files executed throughout this work are included with this supporting information. Moreover, the human readable output of the synthetic chemical information declared in the χDL files is reported below. The software stack required to execute χDL files on a Chemputer platform has been installed and used as previously described.[1] Further information is available from the corresponding author upon request.

3.1 Hardware Integration into the Software Stack

When selecting hardware devices to support automated and autonomous synthesis, accessibility to the programming interface is a key consideration and is always discussed with the manufacturer prior to acquisition. In this work, such interfaces were openly available or readily provided by manufacturers upon purchase of the instrument; however, this level of

access is not universally available, particularly for highly specialized or proprietary equipment. We emphasize that the widespread adoption of standardized digital control languages and open communication protocols is essential to enable scalable, interoperable, and vendor-independent laboratory automation. To ensure compatibility between our automation framework and commercial instruments, we prioritize devices that provide accessible programming interfaces. The adoption of standardized digital control languages and open communication protocols are critical for bridging self-developed automation platforms with commercial or industrial systems.

Utility functions were added to SerialLabware to facilitate reliable and flexible communication with the device hardware (**Figure S10**). These functions abstract low-level device commands into higher-level operations that can be reused and orchestrated from within the broader automation framework. Specifically, functions were implemented to activate or deactivate microwave irradiation and to set or retrieve operational parameters such as irradiation wattage, vessel temperature, and process status flags. This modular approach simplifies hardware control by encapsulating device-specific logic within a standardized interface layer.

SerialLabware communicates with the SAIREM GMS450 microwave generator through its Modbus TCP software interface using the Python package pymodbus 3.11.3, and with the CEM Discover 2.0 microwave generator through its iLink web server using the Python package requests 2.32.5. These connections allow for both direct device control and state monitoring during automated reaction execution. The communication protocols differ in implementation, Modbus TCP providing a register-based industrial control interface, while iLink relies on RESTful HTTP requests. Available commands for each device are defined in the respective instrument manuals and were mapped to the framework through dedicated command dictionaries to ensure reproducibility and maintainability.

The resulting utility functions were then integrated into ChemputerχDL, enabling the specification of hardware-specific steps for microwave-assisted synthesis directly within χDL workflows (**Figure S10**). For instance, the user can instruct the system to irradiate a reaction vessel at a defined wattage or target temperature for a prescribed duration, with automated feedback control ensuring precise execution. This design enables χDL scripts to exert fine-grained, programmable control over commercial hardware, bridging the gap between high-level digital synthesis descriptions and physical laboratory execution.

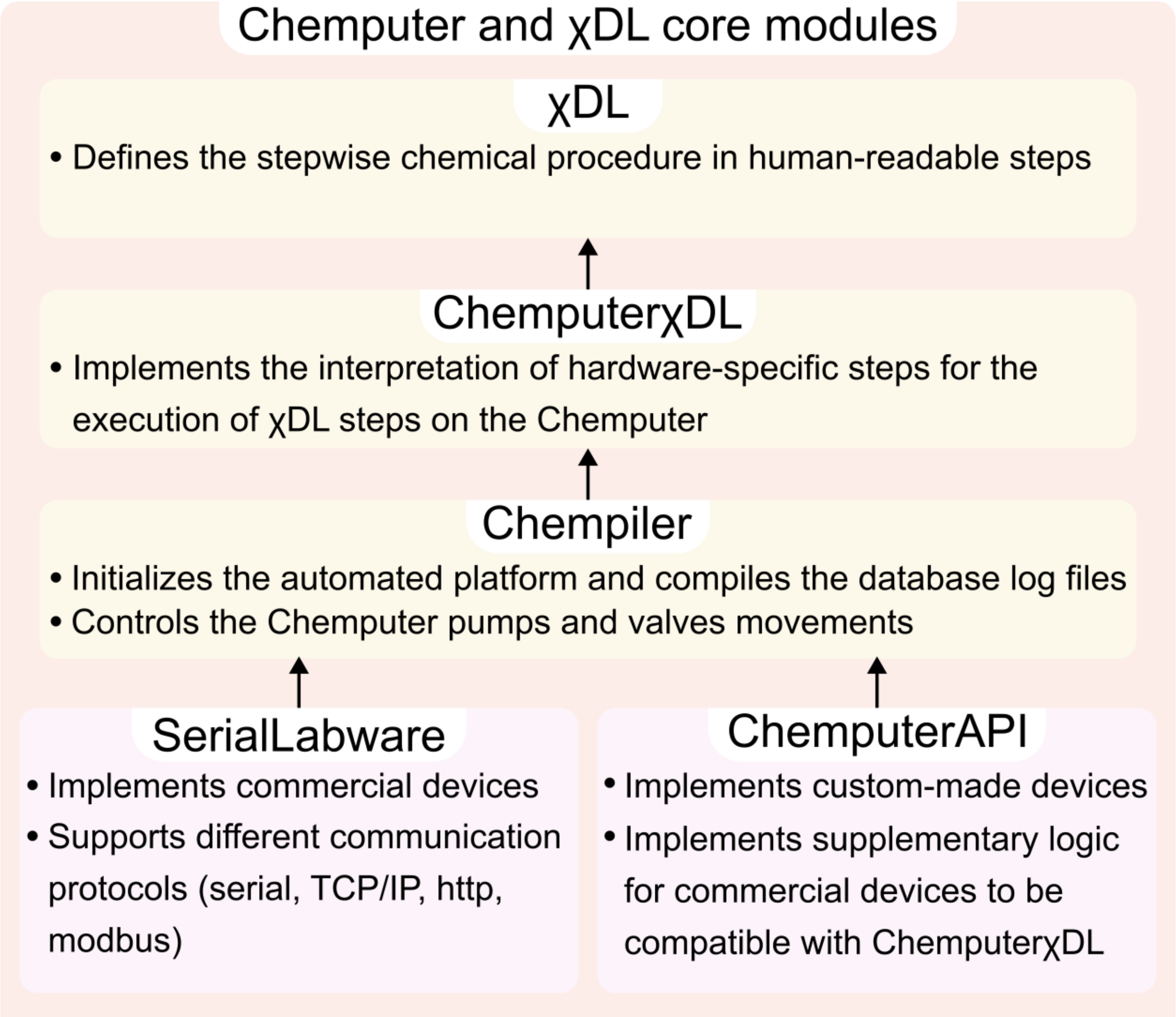

Figure S10. Hierarchy and interdependence of χDL repositories**.**

3.2 Execution of χDL Files for Automated Syntheses

A characteristic example for the execution of a fully automated synthesis procedure on the Chemputer is identified by a χDL file, a graph file, and a Python execution script. The χDL file is written in the XML markup language and saved as a **.xdl** file. Writing and editing of such files is carried out in any preferred editor software (e.g. Visual Studio Code). The graph files are written in the json format and saved as **.json** files. These are created and edited via the ChemIDE web application graphical user interface (GUI; https://croningroup.gitlab.io/chemputer/xdlapp/). Finally, a short Python script is used to finalize the automated execution of χDL files on the Chemputer platform as outlined in **Listing S1**:

Listing S1. General Python script used to execute χDL scripts on the Chemputer platform.

```python
# Importing of the Python libraries needed to execute a XDL synthesis script
# on the Chemputer platform.

from xdl import XDL
import ChemputerAPI
from chempiler import Chempiler
from chemputerxdl import ChemputerPlatform

# Definition of the XDL and graph input files.
experiment_name = "Microwave experiment"
graph_file = experiment_name + ".json"
xdl_file = experiment_name + ".xdl"

# Loading of XDL synthesis script.
# If working with many Blueprints, it is convenient to define a working
# directory where the Blueprint XDL files are stored.

x = XDL(
    xdl_file,
    platform=ChemputerPlatform
)

x.prepare_for_execution(graph_file)

# Connection of the physical hardware through the platform controller
# (Chempiler).

c = Chempiler(
    experiment_name,
    graph_file=graph_file,
    output_dir=experiment_name,
    simulation=False,
    device_modules=[ChemputerAPI],
)
# Execution of the XDL digital synthesis script using the Chempiler.

x.execute(c)
```

The above script can be saved as a **.py** file and directly executed. Alternatively, an interactive Python notebook (such as Jupyter) can be used to execute each step individually, allowing for more flexibility when setting up an automated chemical reaction. All syntheses presented below were executed using a Jupyter notebook.

3.3 χDL blueprints for automated microwave-assisted SPPS

Following reported procedures[2] adapted for microwave-assisted synthesis, the synthetic protocol for SPPS was captured in unambiguous χDL blueprints, each representing one stage of the iterative cycle. The complete SPPS blueprint steps, in human-readable format, showcasing the sequential χDL unit operations executed by the Chemputer platform were the following:

Note: some steps are executed in parallel without waiting for the end of the step preceding them. These are highlighted by an asterisk (*) after the step index.

Note: the general blueprints showcased here contain universal values, highlighted in **bold**, for parameters regarding solvents, amino acids, time, and more synthetic values. During execution, these are specified into the desired property in the χDL file.

Note: in the case a step required manual intervention (i.e. solid additions), this is highlighted in *italics*.

Blueprint: Resin_swell

Step 1: Reset handling by cleaning the backbone with DMF (3 x 3 mL).

Step 2: Add DMF (9 mL) directly to MW_SPPS_Reactor at 100 mL/min.

Step 3: Purge MW_SPPS_Reactor with inert gas for 60 min.

Step 4: Filter contents of MW_SPPS_Reactor, applying vacuum for 50 s.

Blueprint: Deprotection_MW

Step 1: Add 20% piperidine in DMF (9 mL) directly to MW_SPPS_Reactor at 100 mL/min.

Step 2: Start purging MW_SPPS_Reactor with inert gas.

Step 3: Repeat 3 times:

  Microwave MW_SPPS_Reactor with 150 W for 15 s.

  Wait for 45 s.

Step 4: Stop purging MW_SPPS_Reactor with inert gas.

Step 5: Filter contents of MW_SPPS_Reactor, applying vacuum for 80 s.

Step 6: Reset handling by cleaning the backbone with DMF (3 x 3 mL).

Blueprint: Resin_wash

Step 1: Add **wash_solvent** (9 mL) directly to MW_SPPS_Reactor at 100 mL/min.

Step 2: Purge MW_SPPS_Reactor with inert gas for 45 s.

Step 3: Filter contents of MW_SPPS_Reactor, applying vacuum for 30 s.

Step 4: Repeat 3 times:

  Add **wash_solvent** (9 mL) directly to MW_SPPS_Reactor at 100 mL/min.

  Purge MW_SPPS_Reactor with inert gas for 45 s.

  Filter contents of MW_SPPS_Reactor, applying vacuum for 30 s.

Step 5: Add **wash_solvent** (9 mL) directly to MW_SPPS_Reactor at 100 mL/min.

Step 6: Purge MW_SPPS_Reactor with inert gas for 45 s.

Step 7: Filter contents of MW_SPPS_Reactor, applying vacuum for 50 s.

The parameter **wash_solvent** specifies the solvent to use when washing the resin solid support during the different stages of the synthesis. The set default value is DMF, meaning that if no other solvent is specified, DMF will be used.

Blueprint: Coupling_MW

Step 1: **Deprotection_MW**

Step 2: **Resin_wash**

Step 3: Add **amino_acid** (2 mL) directly to MW_SPPS_Reactor at 100 mL/min.

Step 4: Add HATU (2 mL) directly to MW_SPPS_Reactor at 100 mL/min.

Step 5: Add DIPEA (0.5 mL) directly to MW_SPPS_Reactor at 100 mL/min.

Step 6: Start purging MW_SPPS_Reactor with inert.

Step 7: Repeat 5 times:

  Microwave MW_SPPS_Reactor with 150 W for 15 s.

  Wait for 45 s.

Step 8: Stop purging MW_SPPS_Reactor with inert gas.

Step 9: Filter contents of MW_SPPS_Reactor, applying vacuum for 80 s.

Step 10: Reset handling by cleaning the backbone with DMF (3 x 3 mL).

Step 11: **Resin_wash**

The parameter **amino_acid** refers to the amino acid used for this particular coupling step. A unique value must be assigned to each **Coupling** blueprint step as this defines the synthetic operations for peptide synthesis. No default value is given.

Blueprint: Cleavage_and_workup

Step 1: Reset handling by cleaning the backbone with DCM (3 x 3 mL).

Step 2: **Resin_wash** – **wash_solvent** = DCM

Step 3: Dry contents of MW_SPPS_Reactor for 15 min at default pressure.

Step 4: Add TIPS (0.5 mL) directly to MW_SPPS_Reactor at 5 mL/min.

Step 5: Add H_2O (0.5 mL) directly to MW_SPPS_Reactor at 5 mL/min.

Step 6: Add TFA (9 mL) directly to MW_SPPS_Reactor at 20 mL/min.

Step 7*: **Cleavage_mix** for a total of 2 h.

Step 8*: Heat/Chill Precipitating_unit to -20 °C without stirring. Temperature control is continued after the temperature has been reached.

Step 9*: Reset handling by cleaning the backbone with H_2O (3 x 3 mL).

Step 10*: Reset handling by cleaning the backbone with Et_2O (3 x 3 mL).

Step 11*: Add Et_2O (180 mL) directly to Precipitating_unit at 100 mL/min.

Step 12: Transfer all from MW_SPPS_Reactor directly to Precipitating_unit at 20 mL/min, flushing tubing after the transfer.

Step 13: Add TFA (10 mL) directly to MW_SPPS_Reactor at 20 mL/min.

Step 14: Purge MW_SPPS_Reactor with inert gas for 60 s.

Step 15: Transfer all from MW_SPPS_Reactor directly to Precipitating_unit at 20 mL/min, flushing tubing after the transfer.

Step 16*: Purge Precipitating_unit with inert gas for 30 min.

Step 17*: Reset handling by cleaning the backbone with H_2O (3 x 3 mL).

Step 18*: Reset handling by cleaning the backbone with Et_2O (3 x 3 mL).

Step 19: Filter contents of Precipitating_unit, applying vacuum for 30 s, sending filtrate to Supernatant at 100 mL/min.

Step 20: Repeat 3 times:

Add Et_2O (30 mL) directly to Precipitating_unit at 100 mL/min.

Purge Precipitating_unit with inert gas for 5 min.

Filter contents of Precipitating_unit, applying vacuum for 30 s, sending filtrate to Supernatant at 100 mL/min.

Step 21: Dry contents of Precipitating_unit for 10 min at default pressure.

Step 22*: Heat/Chill Precipitating_unit to 20 °C without stirring. Temperature control is stopped once the temperature has been reached.

Step 23*: Reset handling by cleaning the backbone with **peptide_solvent** (3 x 3 mL)..

Step 24: Add **peptide_solvent** (20 mL) directly to Precipitating_unit at 100 mL/min.

Step 25: Purge Precipitating_unit with inert gas for 15 min.

Step 26: Transfer all from Precipitating_unit directly to **collection_flask** at 100 mL/min, flushing tubing after the transfer.

Step 27: Repeat 2 times:

Add **peptide_solvent** (10 mL) directly to Precipitating_unit at 100 mL/min.

Purge Precipitating_unit with inert gas for 2 min.

Transfer all from Precipitating_unit directly to **collection_flask** at 100 mL/min, flushing tubing after the transfer.

Step 28: Reset handling by cleaning the backbone with DCM (3 x 3 mL).

Step 29: Reset handling by cleaning the backbone with DMF (3 x 3 mL).

The blueprint step **Cleavage_mix** is a simple blueprint to allow the gentle sparging of the resin in the cleavage mixture and the parallel execution of the following steps in preparation of peptide precipitation and workup.

The parameter **peptide_solvent** refers to the solvent used to dissolve the peptide after ether workup. This would usually be MeCN/H2O (50:50 v/v) for lyophilization, an adequate solvent for chemical modifications, or a buffer solution as required.

The parameter **collection_flask** refers to the destination vessel after diethyl ether workup. This can be a vial for subsequent lyophilization, a reactor for chemical modifications, or another module as required.

4. Fully automated microwave-assisted chemical synthesis

All procedures discussed in the following sections were carried out in complete laboratory automation. Manual steps included platform preparation prior to execution, steps highlighted in *italics* within the human readable outputs, and transfers between Chemputer, automated flash chromatography system, and analytical instruments.

4.1 Ambient pressure open vessel microwave-assisted O-alkylation (3)

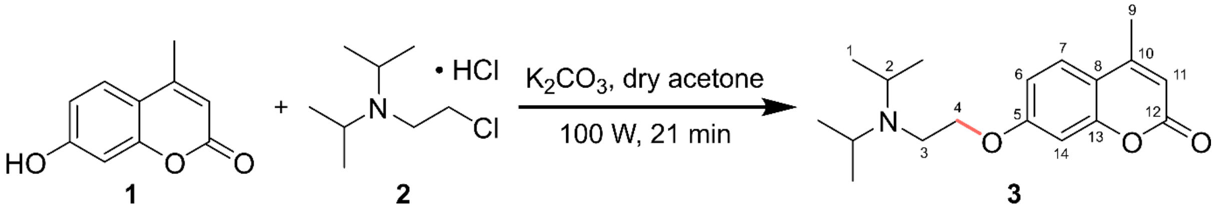

The Chemputer platform was arranged as outlined in the graph file O-alkylation_3-1.json and the synthetic procedure executed according to the χDL file O-alkylation_3-1.xdl included with the supporting information.

The human readable output from the automated procedure is as follows:

Step 1: Set stir rate of MW Reactor to 250 RPM.

Step 2: Set stir rate of Filter 1 to 250 RPM.

Step 3: Add K_2CO_3 (8.2 g) directly to MW Reactor.

Step 4: Add 7-Hydroxy-4-methylcoumarin (2.0 g) directly to MW Reactor.

Step 5: Add 2-Diisopropylaminoethyl chloride hydrochloride (2.3 g) directly to MW Reactor.

Step 6: Reset handling by cleaning the backbone with Acetone (3 x 3 mL).

Step 7: Reset handling by cleaning the backbone with H_2O (3 x 3 mL).

Step 8*: Add H_2O (40 mL) directly to Filter 1 at 100 mL/min with stirring at 250 RPM.

Step 9*: Heat/Chill Filter 1 to 0 °C with stirring at 250 RPM. Temperature control is continued after the temperature has been reached.

Step 10: Set stir rate to 250 RPM and start stirring Filter 1.

Step 11: Reset handling by cleaning the backbone with Dry acetone (3 x 3 mL).

Step 12: Add Dry acetone (50 mL) directly to MW Reactor at 100 mL/min.

Step 13: Set stir rate to 250 RPM and start stirring MW Reactor.

Step 14: Repeat 3 times:

  Microwave MW Reactor for 5 min at a power of 100 W.

  Wait for 2 min.

Step 15: Stir MW Reactor for 2 h at 250 RPM stopping stirring afterwards.

Step 16: Transfer 70 mL from MW Reactor directly to Filter 1 at 100 mL/min, flushing tubing after the transfer.

Step 17: Stir Filter 1 for 30 min at 250 RPM stopping stirring afterwards.

Step 18: Heat/Chill Filter 1 to 5 °C with stirring at 250 RPM. Temperature control is continued after the temperature has been reached.

Step 19: Stir Filter 1 for 5 min at 250 RPM stopping stirring afterwards.

Step 20: Filter contents of Filter 1, applying vacuum for 5 min, without stirring, sending filtrate to Supernatant using standard transfer speeds.

Step 21: Wash solid in Filter 1 with H_2O (2 x 30 mL) without temperature control, with stirring at 250 RPM, applying vacuum for 10 s, sending filtrate to Supernatant.

Step 22: Stop heating/chilling Filter 1.

Step 23*: Dry contents of Filter 1 for 30 min at default pressure without temperature control stopping heating when step finishes.

Step 24*: Reset handling by cleaning the backbone with Acetone (3 x 3 mL).

Step 25: Shut down the platform.

Yield = 1.9 g, 6.3 mmol, 56%.

Spectroscopic data was in agreement with the literature.[3]

^{1}H NMR (600 MHz, $CDCl_3$) δ 7.48 (d, *J* = 8.7 Hz, 1H, H_7), 6.84 (dd, *J* = 8.7, 2.5 Hz, 1H, H_6), 6.81 (d, *J* = 2.5 Hz, 1H, H_{14}), 6.12 (d, *J* = 1.3 Hz, 1H, H_{11}), 3.95 (t, *J* = 7.2 Hz, 2H, H_4), 3.05 (hept, *J* = 6.5 Hz, 2H, H_2), 2.85 (t, *J* = 7.2 Hz, 2H, H_3), 2.39 (d, *J* = 1.2 Hz, 3H, H_9), 1.05 (d, *J* = 6.5 Hz, 12H, H_1).

^{13}C NMR (151 MHz, $CDCl_3$) δ 162.3 (C_5), 161.5 (C_{12}), 155.5 (C_{13}), 152.7 (C_{10}), 125.6 (C_7), 113.6 (C_8), 112.7 (C_6), 112.0 (C_{11}), 101.8 (C_{14}), 70.0 (C_4), 49.8 (C_2), 44.3 (C_3), 21.0 (C_1), 18.8 (C_9).

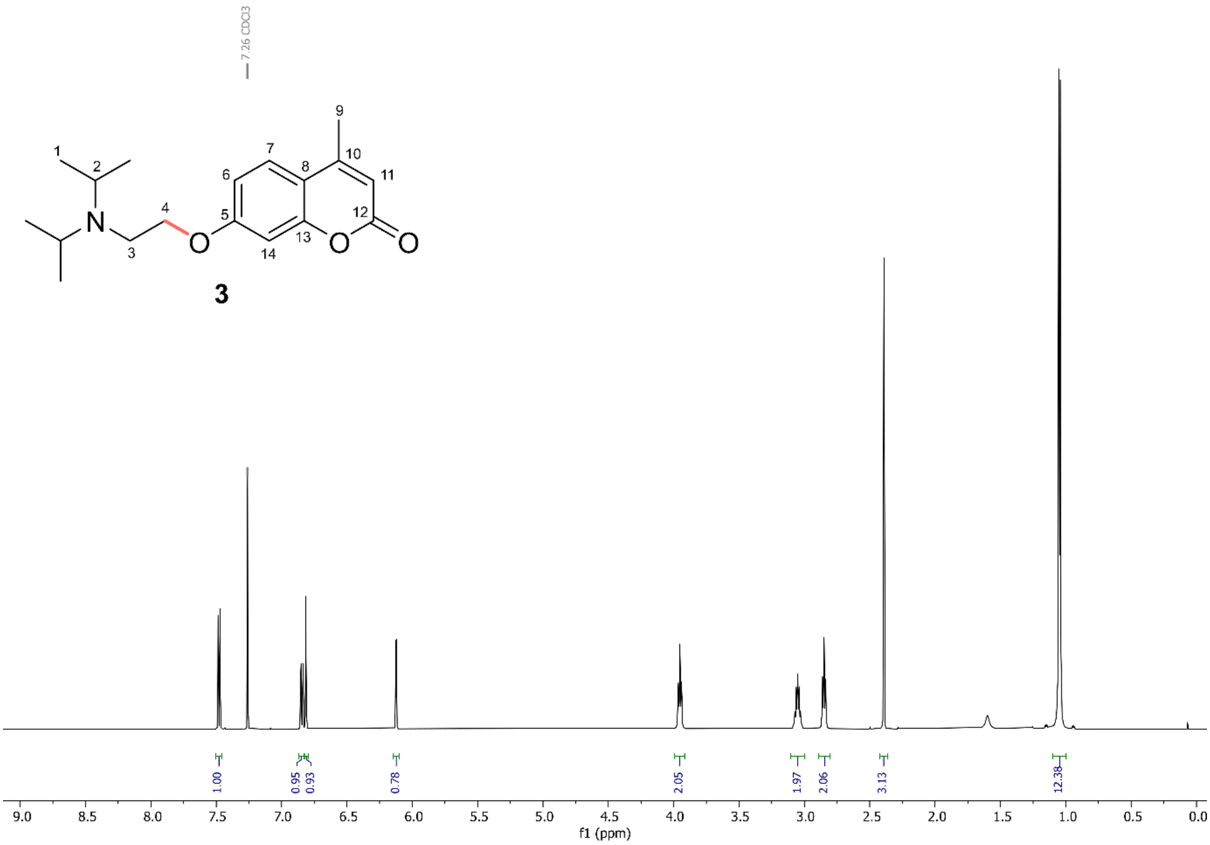

Figure S11. ^{1}H NMR (600 MHz, $CDCl_3$) of **3**.

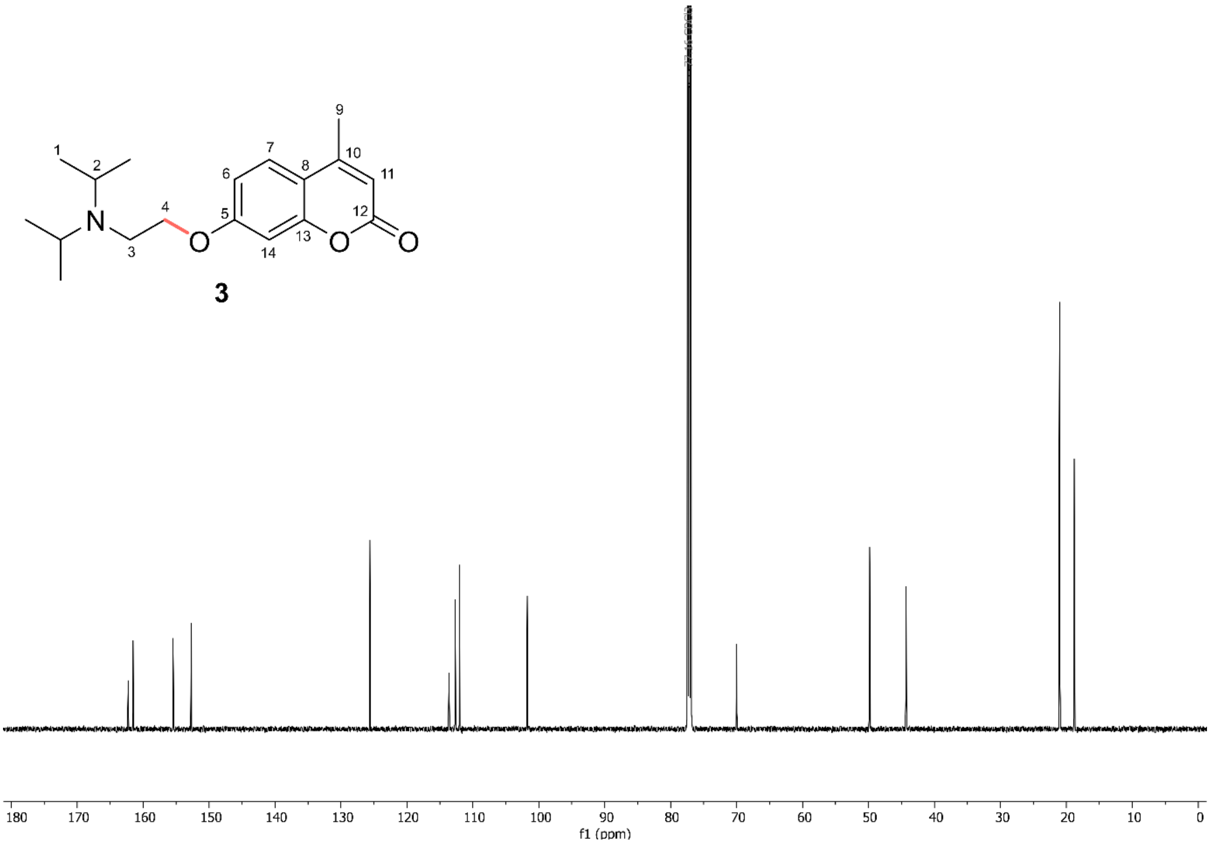

Figure S12. ^{13}C NMR (151 MHz, $CDCl_3$) of **3**.

4.2 Pressurized closed vessel microwave-assisted O-alkylation (3)

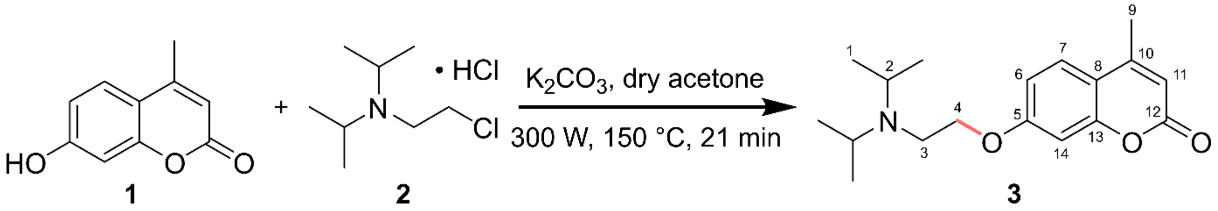

The Chemputer platform was arranged as outlined in the graph file O-alkylation_3-2.json and the synthetic procedure executed according to the χDL file O-alkylation_3-2.xdl included with the supporting information.

The human readable output from the automated procedure is as follows:

Step 1: Set stir rate of MW Reactor to 250 RPM.

Step 2: Set stir rate of Filter 1 to 250 RPM.

Step 3: Add K_2CO_3 *(1.8 g) directly to MW Reactor.*

Step 4: Add 7-Hydroxy-4-methylcoumarin (0.44 g) directly to MW Reactor.

Step 5: Add 2-Diisopropylaminoethyl chloride hydrochloride (0.75 g) directly to MW Reactor.

Step 6: Reset handling by cleaning the backbone with Acetone (3 x 3 mL).

Step 7: Reset handling by cleaning the backbone with H_2O (3 x 3 mL).

Step 8*: Add H_2O (40 mL) directly to Filter 1 at 100 mL/min with stirring at 250 RPM.

Step 9*: Heat/Chill Filter 1 to 0 °C with stirring at 250 RPM. Temperature control is continued after the temperature has been reached.

Step 10: Set stir rate to 250 RPM and start stirring Filter 1.

Step 11: Reset handling by cleaning the backbone with Dry acetone (3 x 3 mL).

Step 12: Add Dry acetone (10 mL) directly to MW Reactor at 100 mL/min.

Step 13: Set stir rate to 900 RPM and start stirring MW Reactor.

Step 14: Microwave MW Reactor for 21 min at a power of 300 W at 150 °C.

Step 15: Stir MW Reactor for 2 h at 900 RPM stopping stirring afterwards.

Step 16: Transfer 30 mL from MW Reactor directly to Filter 1 at 100 mL/min, flushing tubing after the transfer.

Step 17: Stir Filter 1 for 30 min at 250 RPM stopping stirring afterwards.

Step 18: Heat/Chill Filter 1 to 5 °C with stirring at 250 RPM. Temperature control is continued after the temperature has been reached.

Step 19: Stir Filter 1 for 5 min at 250 RPM stopping stirring afterwards.

Step 20: Filter contents of Filter 1, applying vacuum for 5 min, without stirring, sending filtrate to Supernatant using standard transfer speeds.

Step 21: Wash solid in Filter 1 with H_2O (2 x 30 mL) without temperature control, with stirring at 250 RPM, applying vacuum for 10 s, sending filtrate to Supernatant.

Step 22: Stop heating/chilling Filter 1.

Step 23*: Dry contents of Filter 1 for 30 min at default pressure without temperature control stopping heating when step finishes.

Step 24*: Reset handling by cleaning the backbone with Acetone (3 x 3 mL).

Step 25: Shut down the platform.

Yield = 0.48 g, 1.6 mmol, 64%.

Spectroscopic data was in agreement with the literature.[3]

^{1}H NMR (600 MHz, $CDCl_3$) δ 7.48 (d, *J* = 8.8 Hz, 1H, H_7), 6.84 (dd, *J* = 8.8, 2.5 Hz, 1H, H_6), 6.81 (d, *J* = 2.5 Hz, 1H, H_{14}), 6.12 (d, *J* = 1.2 Hz, 1H, H_{11}), 3.95 (t, *J* = 7.2 Hz, 2H, H_4), 3.05 (hept, *J* = 6.5 Hz, 2H, H_2), 2.85 (t, *J* = 7.2 Hz, 2H, H_3), 2.39 (d, *J* = 1.3 Hz, 3H, H_9), 1.05 (d, *J* = 6.5 Hz, 12H, H_1).

^{13}C NMR (151 MHz, $CDCl_3$) δ 162.3 (C_5), 161.5 (C_{12}), 155.5 (C_{13}), 152.7 (C_{10}), 125.6 (C_7), 113.6 (C_8), 112.7 (C_6), 112.0 (C_{11}), 101.7 (C_{14}), 70.0 (C_4), 49.8 (C_2), 44.3 (C_3), 21.0 (C_1), 18.8 (C_9).

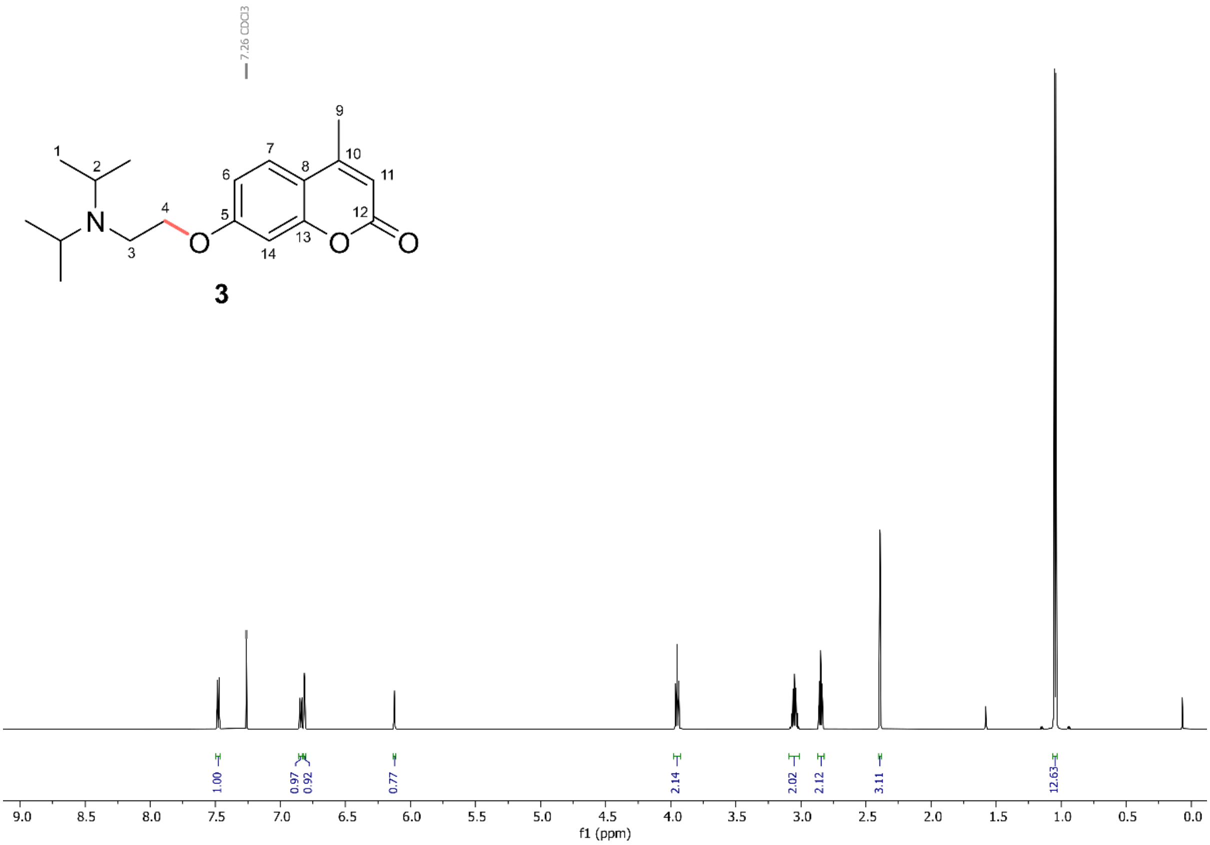

Figure S13. ^{1}H NMR (600 MHz, $CDCl_3$) of **3**.

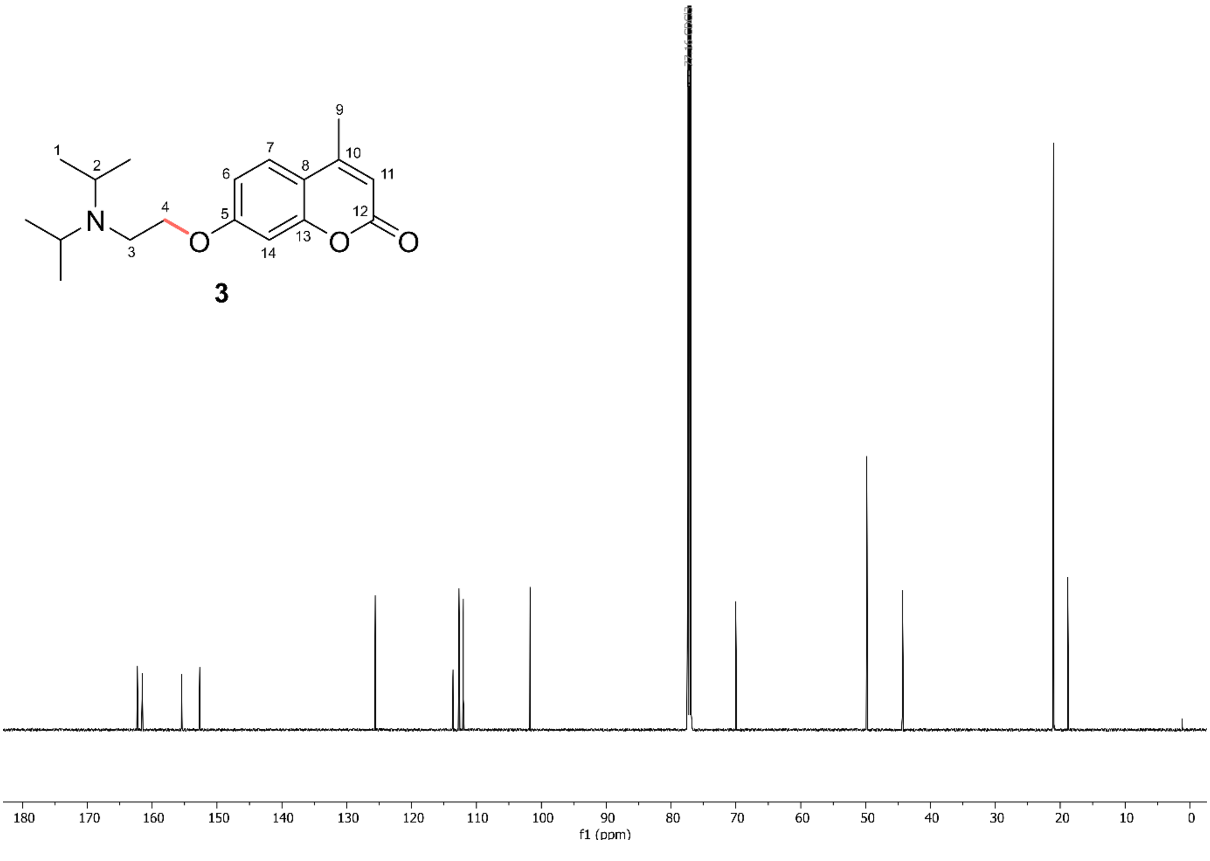

Figure S14. ^{13}C NMR (151 MHz, $CDCl_3$) of **3**.

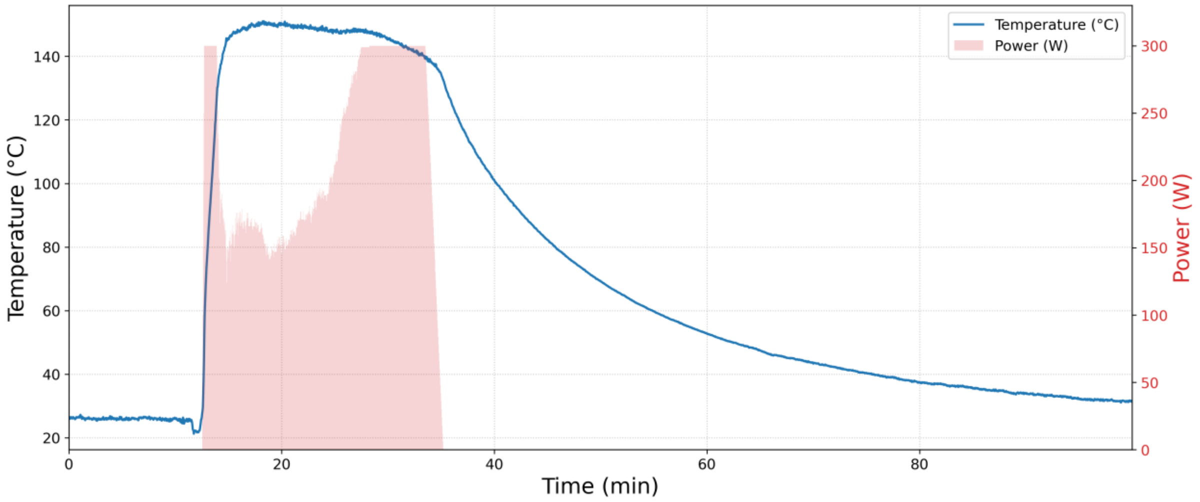

Figure S15. Temperature and power profile over time for the microwave irradiation for the pressurized closed vessel microwave-assisted O-alkylation.

4.3 Ambient pressure open vessel microwave-assisted C–C Suzuki–Miyaura cross-coupling (6)

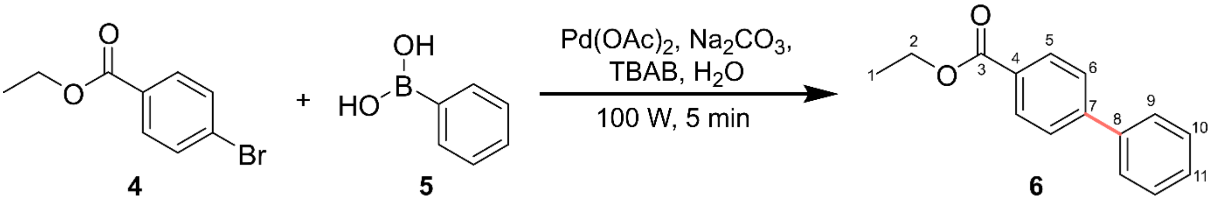

The Chemputer platform was arranged as outlined in the graph file Cross-coupling_6-1.json and the synthetic procedure executed according to the χDL file Cross-coupling_6-1.xdl included with the supporting information. Stock solutions of Na_2CO_3 (1.9 M in H_2O) and TBAB (1.0 M in H_2O) were prepared and loaded as reagents on the Chemputer platform before execution.

The human readable output from the automated procedure is as follows:

Step 1: Set stir rate of Separator 1 to 250 RPM.

Step 2: Set stir rate of Rotovap to 250 RPM.

Step 3: Set stir rate of MW Reactor to 250 RPM.

Step 4: Add Phenylboronic acid (1.2 g) directly to MW Reactor.

Step 5: Add $Pd(OAc)_2$ (9.0 mg) directly to MW Reactor.

Step 6: Reset handling by cleaning the backbone with Acetone (3 x 3 mL).

Step 7: Reset handling by cleaning the backbone with H_2O (3 x 3 mL).

Step 8: Add Ethyl 4-bromobenzoate (1.7 mL) directly to MW Reactor at 100 mL/min.

Step 9: Start stirring MW Reactor.

Step 10: Add Na_2CO_3 (28 mL) directly to MW Reactor at 100 mL/min.

Step 11: Add TBAB (2 mL) directly to MW Reactor at 100 mL/min.

Step 12: Add H_2O (4 mL) directly to MW Reactor at 100 mL/min.

Step 13: Reset handling by cleaning the backbone with H_2O (3 x 3 mL).

Step 14: Microwave MW Reactor for 5 min at a power of 100 W.

Step 15: Stir MW Reactor for 10 min at 250 RPM stopping stirring afterwards.

Step 16: Transfer 50 mL from MW Reactor directly to Separator 1, rinsing with Et_2O (50 mL) at 100 mL/min, without flushing tubing after the transfer.

Step 17: Add H_2O (30 mL) directly to Separator 1 at 100 mL/min.

Step 18: Extract two-phase mixture in Separator 1 without adding solvent. Transfer waste phase (bottom) to vessel Buffer flask 1, and product phase (top) directly to Product 1.

Step 19: Extract contents of Buffer flask 1 with Et_2O (2 x 25 mL). Transfer waste phase (bottom) to vessel Buffer flask 1, and product phase (top) directly to Product 1.

Step 20: Reset handling by cleaning the backbone with Acetone (3 x 3 mL).

Step 21: Reset handling by cleaning the backbone with Et_2O (3 x 3 mL).

Step 22: Transfer 120 mL from Product 1 through $MgSO_4$_Cartridge to Rotovap at 100 mL/min, without flushing tubing after the transfer.

Step 23: Reset handling by cleaning the backbone with Acetone (3 x 3 mL).

Step 24: Evaporate contents of Rotovap with default pressure control at temperature 40 °C in auto mode.

Step 25: Evaporate contents of Rotovap with pressure 60 mbar at temperature 40 °C for 60 min.

Step 26: Shut down the platform.

The crude material was then transferred to an automated flash chromatography system for purification on silica gel (2% ethyl acetate in hexane 1 column volume, 2-10% ethyl acetate in hexane 20 column volumes) to afford **6**.

Yield = 1.1 g, 4.9 mmol, 49%.

Spectroscopic data was in agreement with the literature.[4]

^{1}H NMR (600 MHz, $CDCl_3$) δ 8.15 (d, *J* = 8.4 Hz, 2H, H_5), 7.67 (d, *J* = 8.4 Hz, 2H, H_6), 7.63 (d, *J* = 7.4 Hz, 2H, H_9), 7.47 (t, *J* = 7.4 Hz, 2H, H_{10}), 7.41 (t, *J* = 7.4 Hz, 1H, H_{11}), 4.43 (q, *J* = 7.1 Hz, 2H, H_2), 1.44 (t, *J* = 7.1 Hz, 3H, H_1).

^{13}C NMR (151 MHz, $CDCl_3$) δ 166.5 (C_3), 145.5 (C_7), 140.0 (C_8), 130.1 (C_5), 129.3 (C_4), 128.9 (C_{10}), 128.1 (C_{11}), 127.3 (C_9), 127.0 (C_6), 61.0 (C_2), 14.4 (C_1).

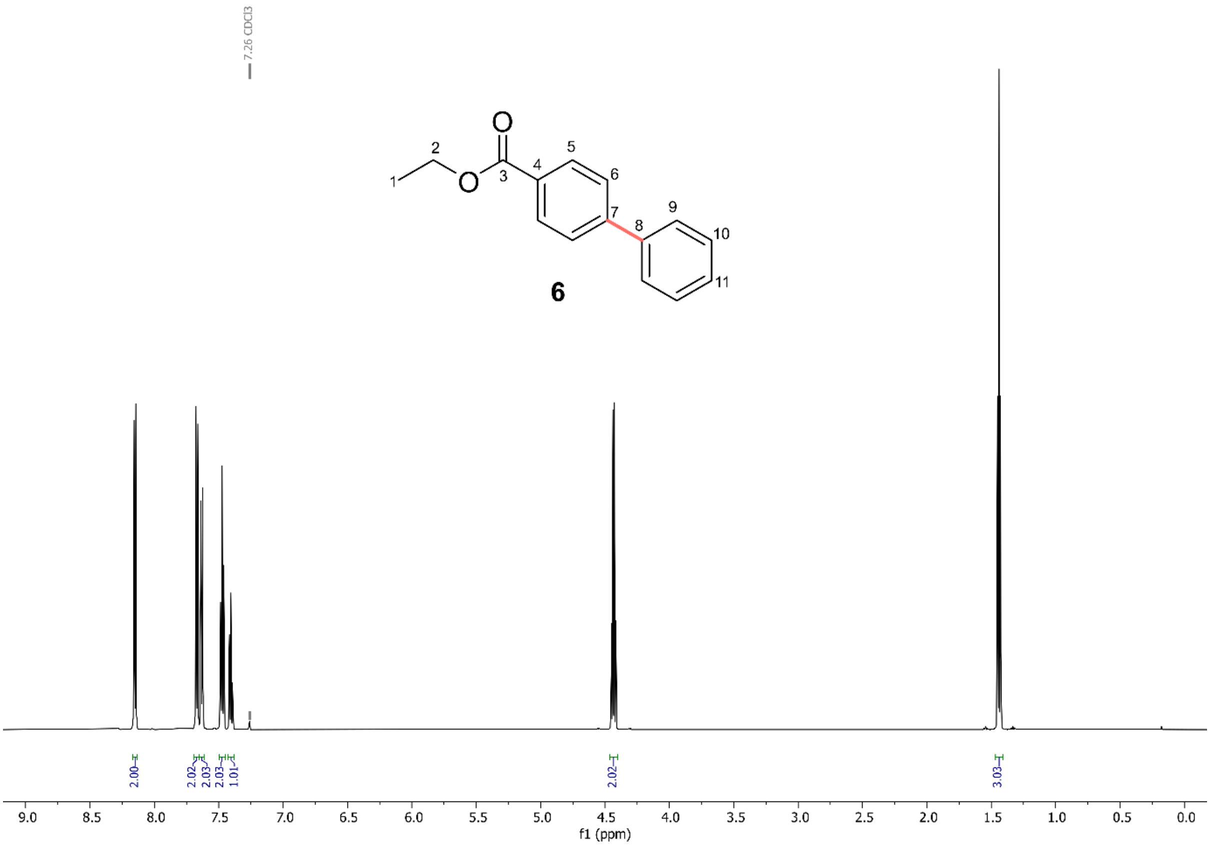

Figure S16. ^{1}H NMR (600 MHz, $CDCl_3$) of **6**.

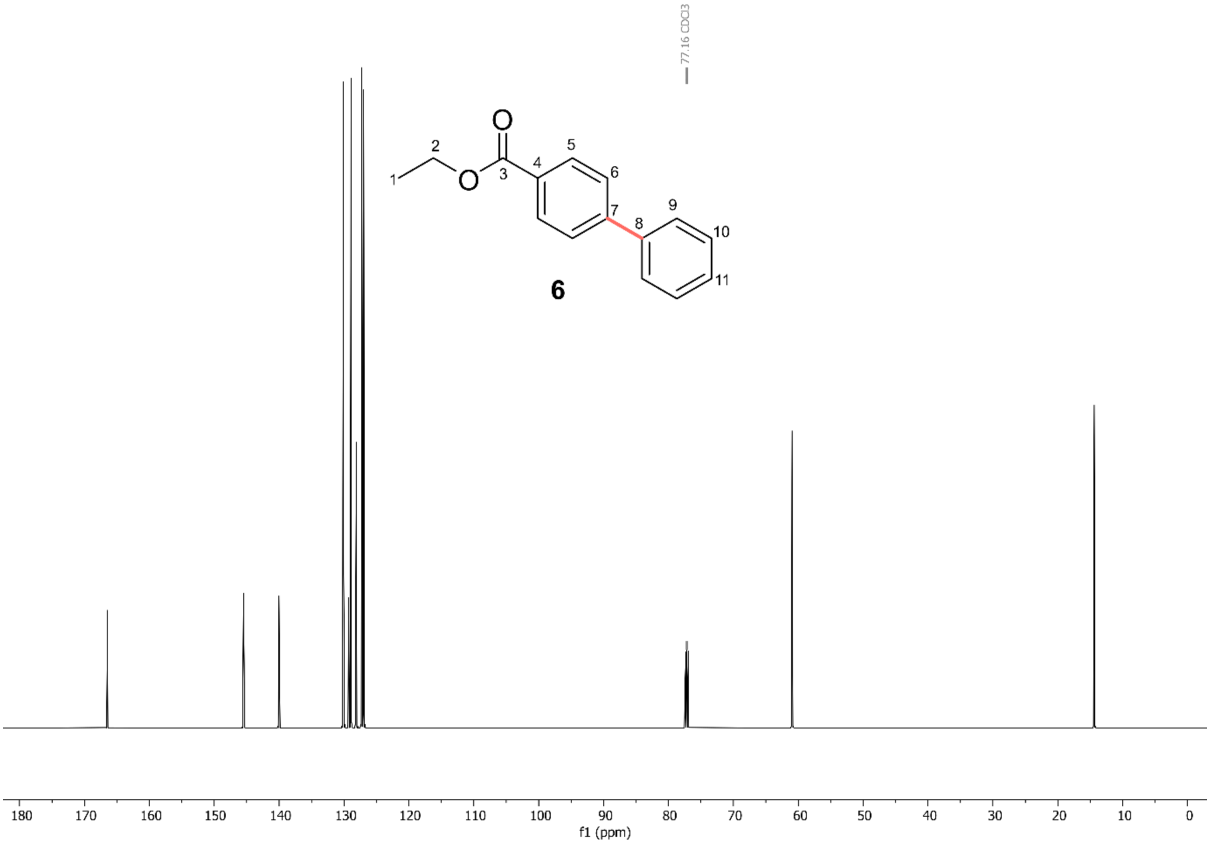

Figure S17. ^{13}C NMR (151 MHz, $CDCl_3$) of **6**.

4.4 Pressurized closed vessel microwave-assisted C–C Suzuki–Miyaura cross-coupling (6)

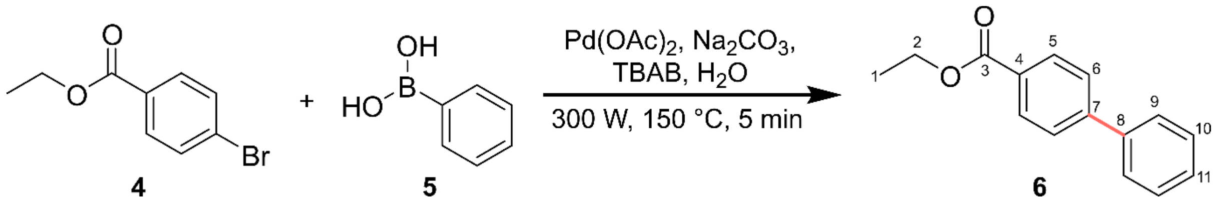

The Chemputer platform was arranged as outlined in the graph file Cross-coupling_6-2.json and the synthetic procedure executed according to the χDL file Cross-coupling_6-2.xdl included with the supporting information. Stock solutions of Na_2CO_3 (1.9 M in H_2O) and TBAB (1.0 M in H_2O) were prepared and loaded as reagents on the Chemputer platform before execution.

The human readable output from the automated procedure is as follows:

Step 1: Set stir rate of Separator 1 to 250 RPM.

Step 2: Set stir rate of Rotovap to 250 RPM.

Step 3: Set stir rate of MW Reactor to 250 RPM.

Step 4: Add Phenylboronic acid (1.2 g) directly to MW Reactor.

Step 5: Add $Pd(OAc)_2$ (9.0 mg) directly to MW Reactor.

Step 6: Reset handling by cleaning the backbone with Acetone (3 x 3 mL).

Step 7: Reset handling by cleaning the backbone with H_2O (3 x 3 mL).

Step 8: Add Ethyl 4-bromobenzoate (1.7 mL) directly to MW Reactor at 100 mL/min.

Step 9: Set stir rate to 500 RPM and start stirring MW Reactor.

Step 10: Add Na_2CO_3 (28 mL) directly to MW Reactor at 100 mL/min.

Step 11: Add TBAB (2 mL) directly to MW Reactor at 100 mL/min.

Step 12: Add H_2O (4 mL) directly to MW Reactor at 100 mL/min.

Step 13: Reset handling by cleaning the backbone with H_2O (3 x 3 mL).

Step 14: Microwave MW Reactor for 5 min at a power of 300 W at 150 °C.

Step 15: Stir MW Reactor for 10 min at 250 RPM stopping stirring afterwards.

Step 16: Transfer 50 mL from MW Reactor directly to Separator 1, rinsing with Et_2O (50 mL) at 100 mL/min, without flushing tubing after the transfer.

Step 17: Add H_2O (30 mL) directly to Separator 1 at 100 mL/min.

Step 18: Extract two-phase mixture in Separator 1 without adding solvent. Transfer waste phase (bottom) to vessel Buffer flask 1, and product phase (top) directly to Product 1.

Step 19: Extract contents of Buffer flask 1 with Et_2O (2 x 25 mL). Transfer waste phase (bottom) to vessel Buffer flask 1, and product phase (top) directly to Product 1.

Step 20: Reset handling by cleaning the backbone with Acetone (3 x 3 mL).

Step 21: Reset handling by cleaning the backbone with Et_2O (3 x 3 mL).

Step 22: Transfer 120 mL from Product 1 through $MgSO_4$_Cartridge to Rotovap at 100 mL/min, without flushing tubing after the transfer.

Step 23: Reset handling by cleaning the backbone with Acetone (3 x 3 mL).

Step 24: Evaporate contents of Rotovap with default pressure control at temperature 40 °C in auto mode.

Step 25: Evaporate contents of Rotovap with pressure 60 mbar at temperature 40 °C for 60 min.

Step 26: Shut down the platform.

The crude material was then transferred to an automated flash chromatography system for purification on silica gel (2% ethyl acetate in hexane 1 column volume, 2-10% ethyl acetate in hexane 20 column volumes) to afford **6**.

Yield = 1.3 g, 5.7 mmol, 57%.

Spectroscopic data was in agreement with the literature.[4]

^{1}H NMR (600 MHz, $CDCl_3$) δ 8.15 (d, J = 8.4 Hz, 2H, H_5), 7.67 (d, J = 8.4 Hz, 2H, H_6), 7.63 (d, J = 7.3 Hz, 2H, H_9), 7.47 (t, J = 7.3 Hz, 2H, H_{10}), 7.41 (t, J = 7.3 Hz, 1H, H_{11}), 4.43 (q, J = 7.2 Hz, 2H, H_2), 1.44 (t, J = 7.2 Hz, 3H, H_1).

^{13}C NMR (151 MHz, $CDCl_3$) δ 166.5 (C_3), 145.5 (C_7), 140.0 (C_8), 130.1 (C_5), 129.3 (C_4), 128.9 (C_{10}), 128.1 (C_{11}), 127.3 (C_9), 127.0 (C_6), 60.9 (C_2), 14.4 (C_1).

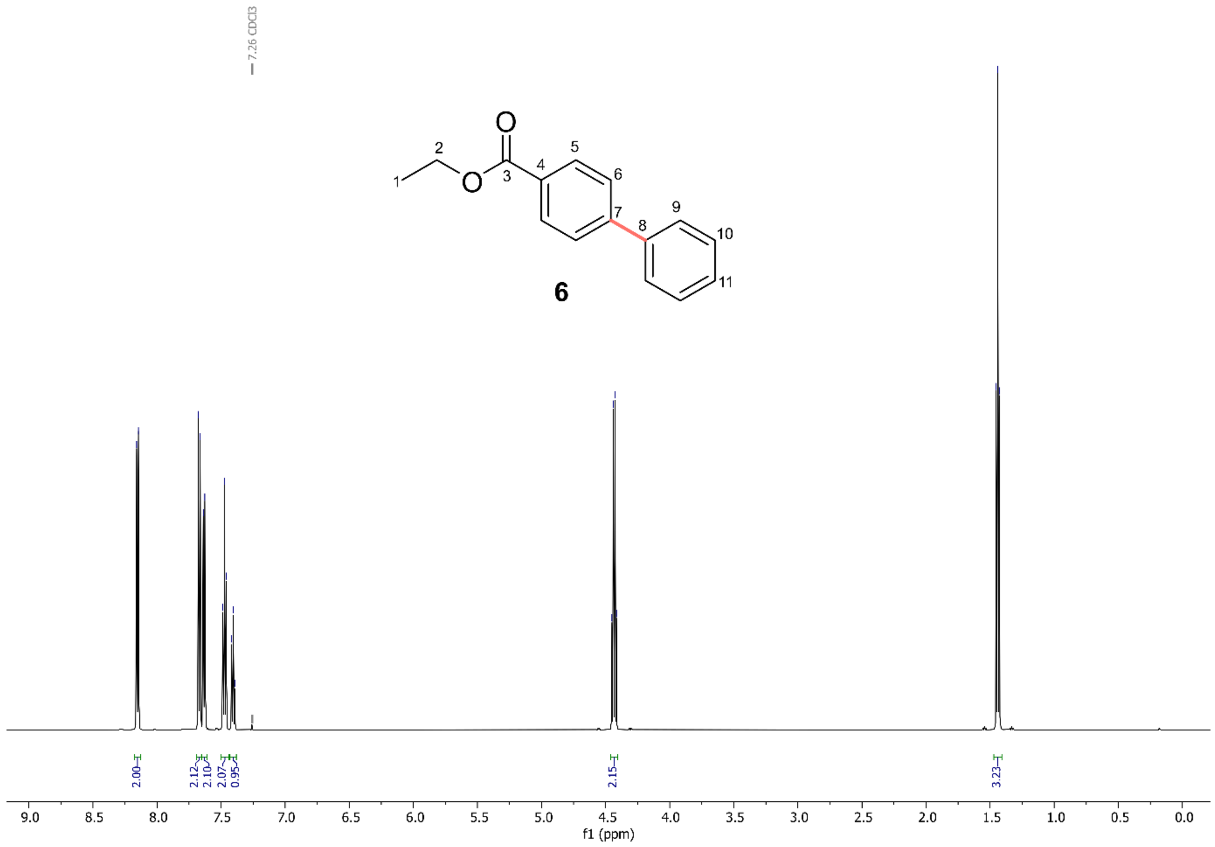

Figure S18. ^{1}H NMR (600 MHz, $CDCl_3$) of **6**.

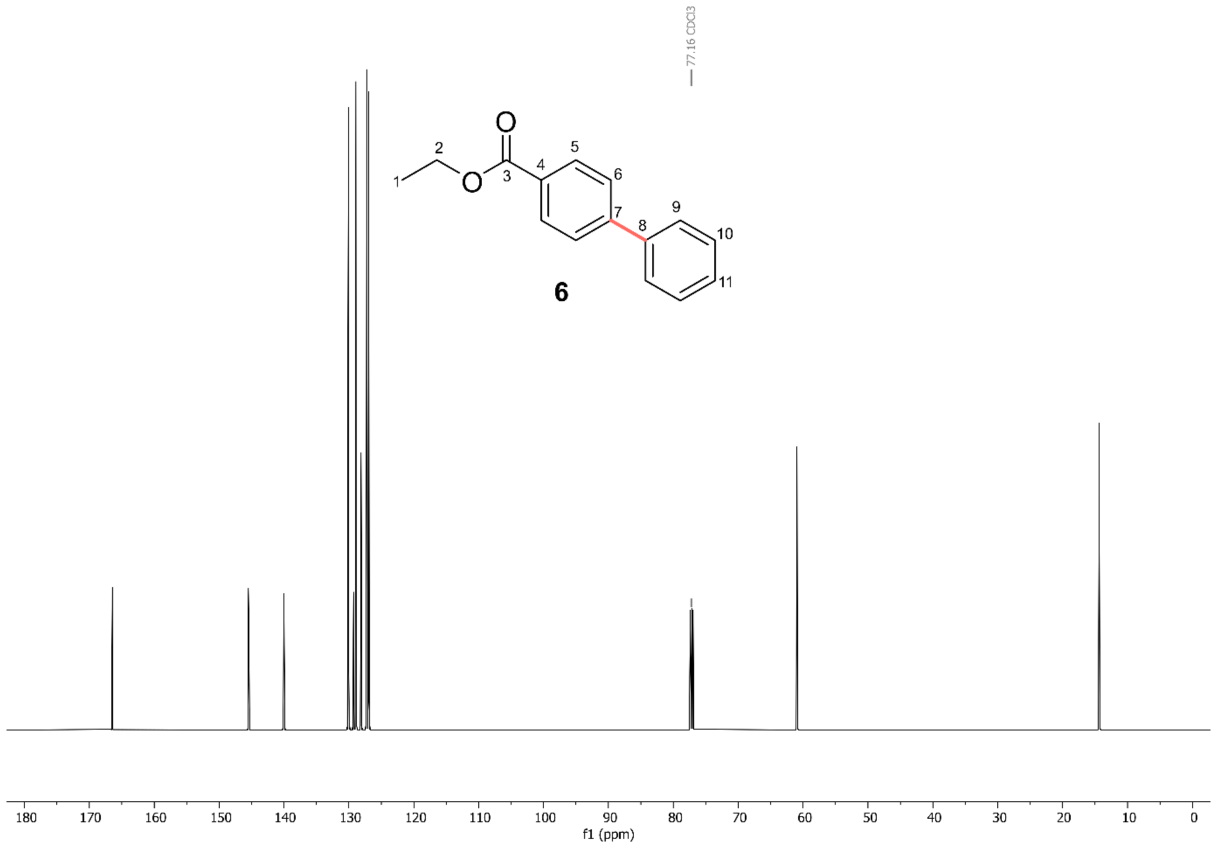

Figure S19. ^{13}C NMR (151 MHz, $CDCl_3$) of **6**.

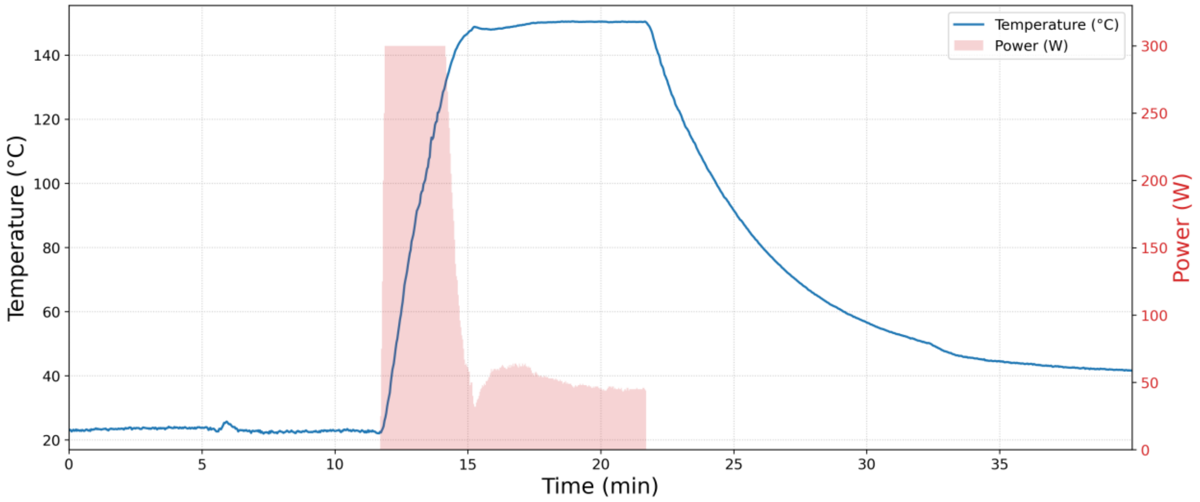

Figure S20. Temperature and power profile over time for the microwave irradiation for the pressurized closed vessel microwave-assisted C–C Suzuki–Miyaura cross-coupling.

4.5 Ambient pressure open vessel microwave-assisted ring-closing metathesis (RCM) (10)

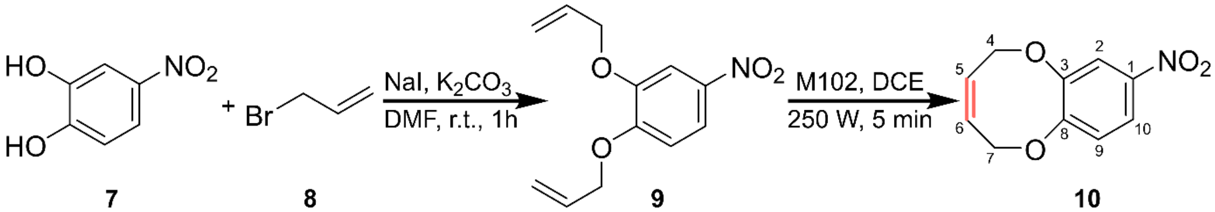

7 **8** **9** **10**

The Chemputer platform was arranged as outlined in the graph file RCM_10.json and the synthetic procedure executed according to the χDL file RCM_10.xdl included with the supporting information. Stock solutions of 4-nitrocatechol (0.5 M in DMF) and allyl bromide (0.5 M in DMF) were prepared and loaded as reagents on the Chemputer platform before execution.

The human readable output from the automated procedure is as follows:

Step 1: Set stir rate of Separator 1 to 250 RPM.

Step 2: Set stir rate of Rotovap to 250 RPM.

Step 3: Set stir rate of MW Reactor to 250 RPM.

Step 4: Set stir rate of Filter 1 to 250 RPM.

Step 5: Add M102 (82 mg) directly to Flask M102 at 100 mL/min.

Step 6: Evacuate Flask M102 and refill with inert gas 3 times, using a vacuum pressure of 50 mbar, waiting 3 min after evacuating and 1 s after refilling with inert gas.

Step 7: Add NaI (15 mg) directly to Filter 1.

Step 8: Add K_2CO_3 (415 mg) directly to Filter 1.

Step 9: Reset handling by cleaning the backbone with DMF (3 x 3 mL).

Step 10: Add 4-Nitrocatechol (2 mL) directly to Filter 1 at 100 mL/min.

Step 11: Add Allyl bromide (5 mL) directly to Filter 1 at 100 mL/min.

Step 12: Add DMF (3 mL) directly to Filter 1 at 100 mL/min.

Step 13*: Stir Filter 1 for 60 min at 250 RPM stopping stirring afterwards.

Step 14*: Reset handling by cleaning the backbone with DMF (3 x 3 mL).

Step 15: Transfer all from Filter 1 directly to Separator 1 at 100 mL/min, flushing tubing after the transfer.

Step 16: Add H_2O (35 mL) directly to Separator 1 at 100 mL/min.

Step 17: Extract contents of Separator 1 with Et_2O (3 x 30 mL). Transfer waste phase (bottom) to vessel Buffer flask 1, and product phase (top) directly to Product 1.

Step 18: Wash contents of Product 1 with H_2O (2 x 30 mL). Transfer waste phase (bottom) to vessel Buffer flask 1, and product phase (top) directly to Separator 1.

Step 19: Wash contents of Separator 1 with Aq LiCl (2 x 30 mL). Transfer waste phase (bottom) to vessel Buffer flask 1, and product phase (top) directly to Separator 1.

Step 20: Transfer all from Separator 1 through $MgSO_4$_Cartridge to Rotovap at 100 mL/min, flushing tubing after the transfer.

Step 21: Add Et_2O (30 mL) through $MgSO_4$_Cartridge to Rotovap at 100 mL/min.

Step 22: Evaporate contents of Rotovap with default pressure control at temperature 40 °C in auto mode.

Step 23: Evaporate contents of Rotovap with pressure 60 mbar at temperature 40 °C for 60 min.

Step 24: Reset handling by cleaning the backbone with DCE (3 x 3 mL).

Step 25: Transfer 10 mL from Argon directly to Waste_6 at 100 mL/min, without flushing tubing after the transfer.

Step 26: Reset handling by cleaning the backbone with DCE (3 x 3 mL).

Step 27: Transfer 10 mL from Argon directly to Waste_6 at 100 mL/min, without flushing tubing after the transfer.

Step 28: Start purging MW Reactor with inert gas.

Step 29: Repeat 2 times:

Add DCE (50 mL) directly to Rotovap at 100 mL/min.

Transfer 70 mL from Rotovap directly to MW Reactor at 100 mL/min, flushing tubing after the transfer.

Step 30: Add DCE (130 mL) directly to MW Reactor at 100 mL/min.

Step 31: Repeat 2 times:

Add DCE (10 mL) directly to Flask M102 at 20 mL/min.

Transfer 20 mL from Flask M102 directly to MW Reactor at 100 mL/min, flushing tubing after the transfer.

Step 32: Set stir rate to 250 RPM and start stirring MW Reactor.

Step 33: Repeat 5 times:

Microwave MW Reactor for 60 s at a power of 250 W.

Wait for 20 s.

Step 34: Stir MW Reactor for 10 min at 250 RPM stopping stirring afterwards.

Step 35: Stop purging MW Reactor with inert gas.

Step 36: Transfer 200 mL from MW Reactor directly to Rotovap at 100 mL/min, flushing tubing after the transfer.

Step 37: Evaporate contents of Rotovap with default pressure control at temperature 40 °C in auto mode.

Step 38: Transfer 100 mL from MW Reactor directly to Rotovap at 100 mL/min, flushing tubing after the transfer.

Step 39: Evaporate contents of Rotovap with default pressure control at temperature 40 °C in auto mode.

Step 40: Evaporate contents of Rotovap with pressure 60 mbar at temperature 40 °C for 60 min.

Step 41: Shut down the platform.

The crude material was then transferred to an automated flash chromatography system for purification on silica gel (0-2% ethyl acetate in hexane 1 column volume, 2-10% ethyl acetate in hexane 5 column volumes, 10% ethyl acetate in hexane 6 column volumes, 10-20% ethyl acetate in hexane 5 column volumes, 20% ethyl acetate in hexane 3 column volumes) to afford **10**.

Yield = 87 mg, 0.42 mmol, 42%.

Spectroscopic data was in general accordance with literature analogues.[5]

^{1}H NMR (600 MHz, $CDCl_3$) δ 7.94 (d, *J* = 2.7 Hz, 1H, H_2), 7.91 (dd, *J* = 9.0, 2.7 Hz, 1H, H_{10}), 6.99 (d, *J* = 9.0 Hz, 1H, H_9), 5.97 – 5.91 (m, 2H, $H_{5,6}$), 5.11 (dd, *J* = 4.3, 2.0 Hz, 2H, H_7), 4.88 (d, *J* = 2.0 Hz, 2H, H_4).

^{13}C NMR (151 MHz, $CDCl_3$) δ 155.3 (C_8), 147.2 (C_3), 142.5 (C_1), 132.9 ($C_{5/6}$), 126.2 ($C_{5/6}$), 121.7 (C_9), 121.1 (C_{10}), 120.9 (C_2), 74.4 (C_7), 67.5 (C_4).

HPLC-UV/vis retention time 16.44 min, 98% purity measured at 254 nm.

ESI-MS calculated *m/z* for $C_{10}H_{10}NO_4^+$ $[M+H]^+$ 208.06, found *m/z* 208.06.

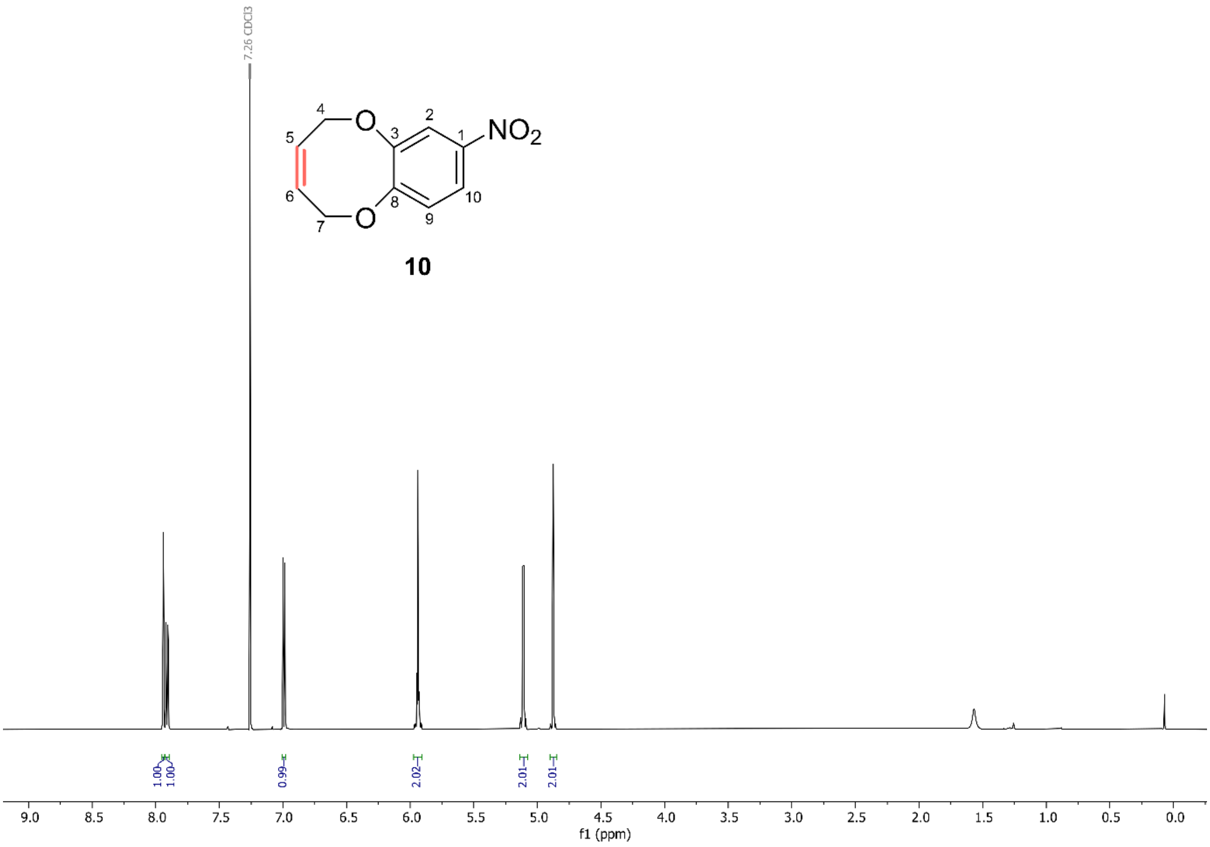

Figure S21. ^{1}H NMR (600 MHz, $CDCl_3$) of **10**.

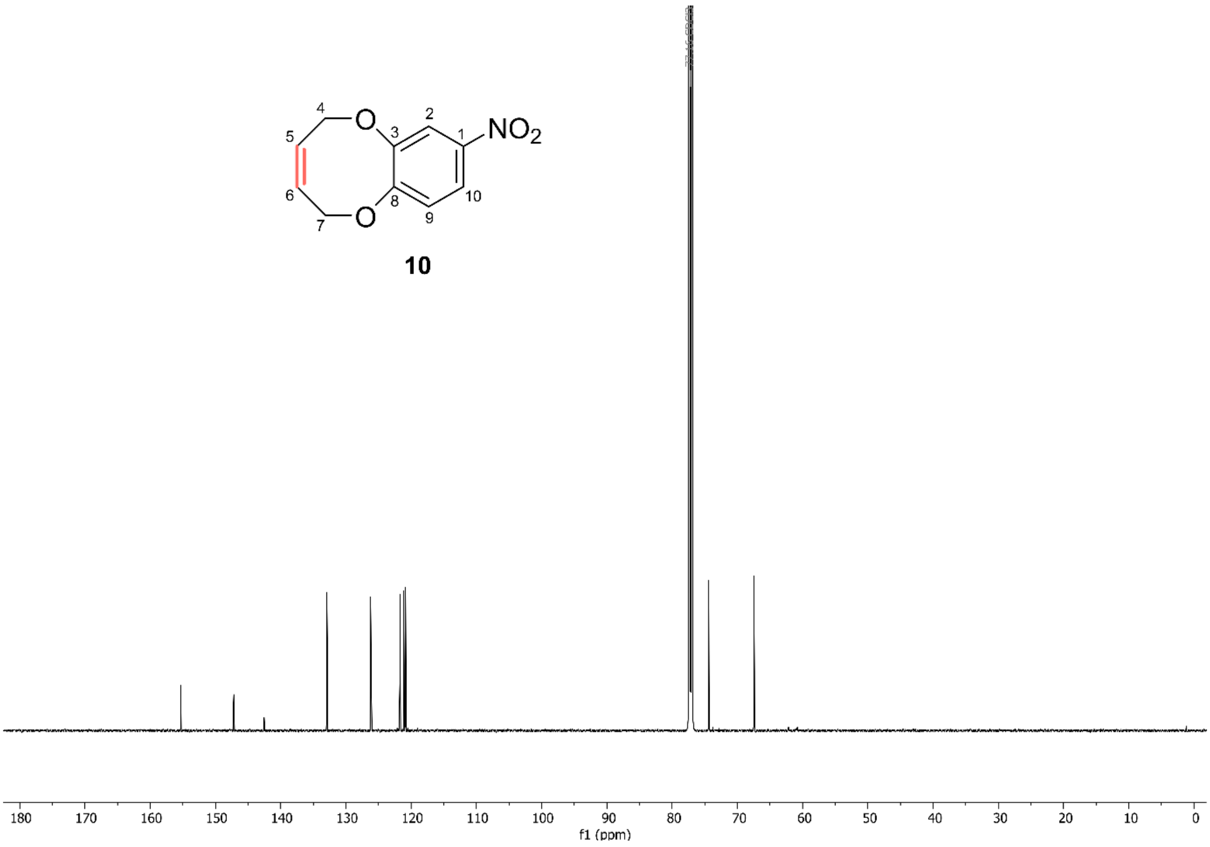

Figure S22. ^{13}C NMR (151 MHz, $CDCl_3$) of **10**.

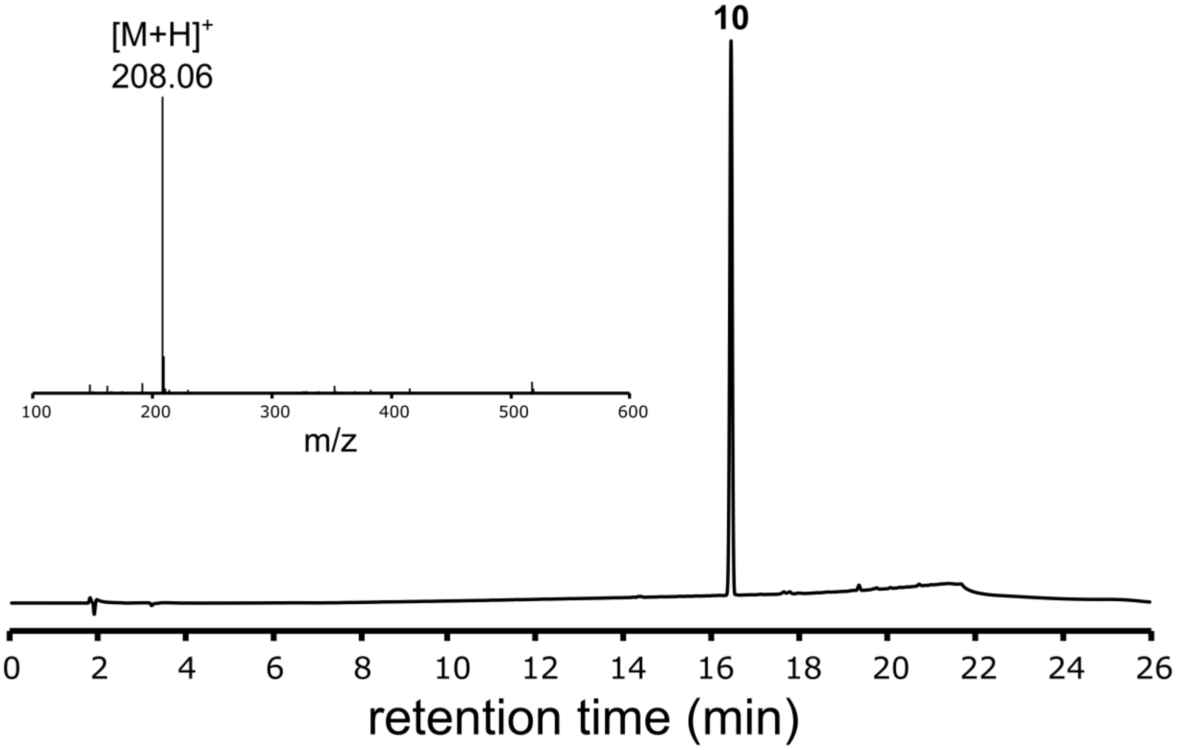

Figure S23. RP-HPLC trace (254 nm, 19 min 0–80% MeCN gradient) and ESI-MS spectra (inlet) of **10**.

4.6 Ambient pressure open vessel microwave-assisted SPPS (13)

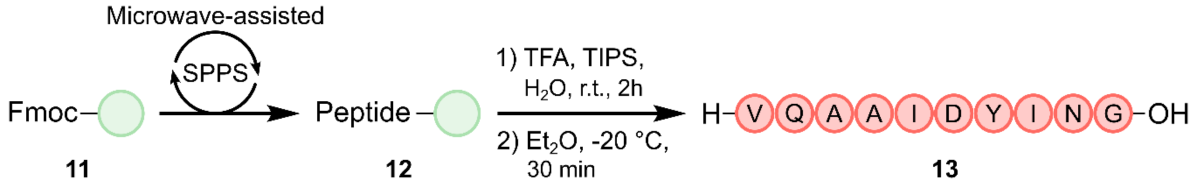

The Chemputer platform was arranged as outlined in the graph file SPPS_13.json and the synthetic procedure executed according to the χDL file SPPS_13.xdl included with the supporting information. The protocol made extensive use of the χDL blueprints highlighted in **Supporting Information 2.4**. Stock solutions of Fmoc protected amino acids (0.5 M in DMF) and HATU (0.5 M in DMF) were prepared and loaded as reagents on the Chemputer platform before execution.

The human readable output from the automated procedure is as follows:

Step 1: Add Fmoc-Gly-Wang resin (278 mg) directly to MW_SPPS_Reactor.

Step 2: **Resin_swell**

Step 3: **Coupling_MW** – amino_acid = Asn

Step 4: **Coupling_MW** – amino_acid = Ile

Step 5: **Coupling_MW** – amino_acid = Tyr

Step 6: **Coupling_MW** – amino_acid = Asp

Step 7: **Coupling_MW** – amino_acid = Ile

Step 8: **Coupling_MW** – amino_acid = Ala

Step 9: **Coupling_MW** – amino_acid = Ala

Step 10: **Coupling_MW** – amino_acid = Gln

Step 11: **Coupling_MW** – amino_acid = Val

Step 12: **Deprotection_MW**

Step 13: **Resin_wash**

Step 14: **Cleavage_and_workup** – peptide_solvent = MeCN/H2O (50:50 v/v), collection_flask = Product

Step 15: Shut down the platform.

Yield = 49 mg, 0.04 mmol, 32%.

HPLC-UV/vis retention time 16.78 min, 76% purity measured at 280 nm.

ESI-MS calculated *m/z* for $C_{47}H_{75}N_{12}O_{16}^+$ [M+H]$^+$ 1063.54, found *m/z* 1063.54.

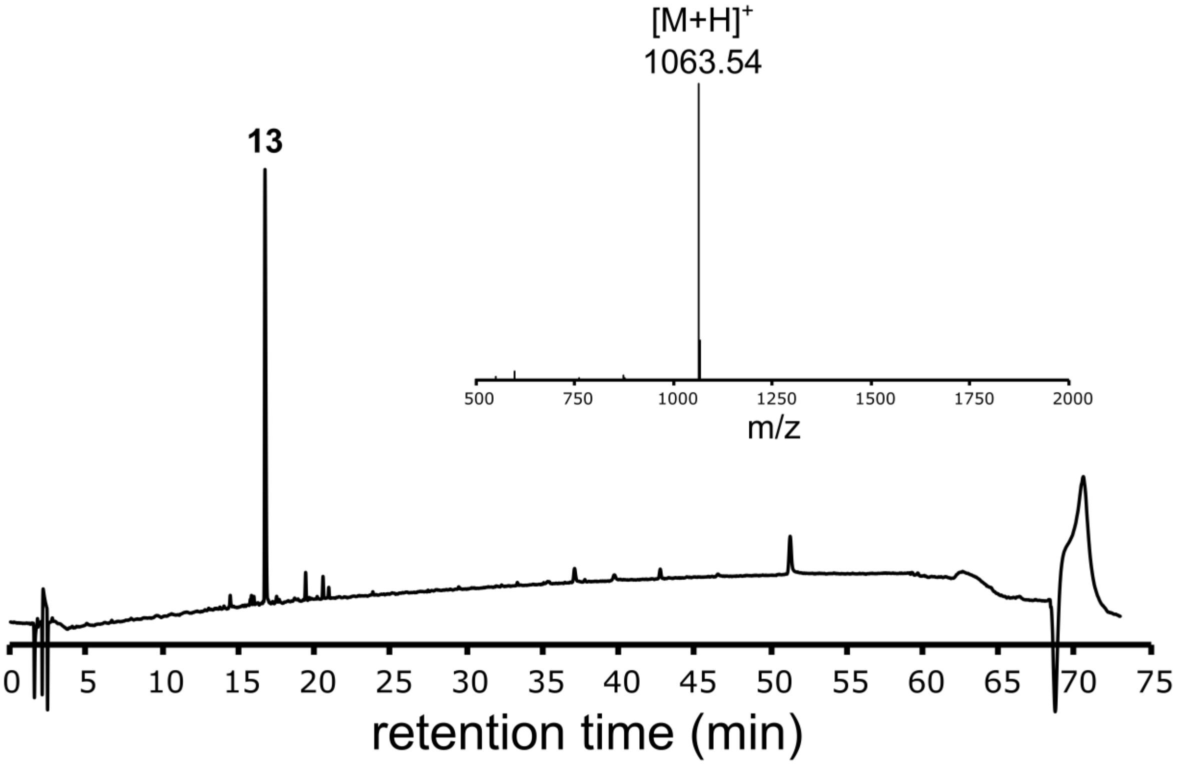

Figure S24. RP-HPLC trace (280 nm, 60 min 0–80% MeCN gradient) and ESI-MS spectra (inlet) of **13**.

5. References

[1] S. Rohrbach, M. Šiaučiulis, G. Chisholm, P.-A. Pirvan, M. Saleeb, S. H. M. Mehr, E. Trushina, A. I. Leonov, G. Keenan, A. Khan, A. Hammer, L. Cronin, *Science* **2022**, *377*, 172–180.

[2] J. Zero, T. J. Tyler, L. Cronin, *Nat. Commun.* **2025**, *16*, 7322.

[3] J. Trykowska, E. Hejchman, I. Wolska, D. Maciejewska, *J. Mol. Struct.* **2009**, *930*, 195–200.

[4] H. Zhou, P. Mukherjee, R. Liu, E. Evrard, D. Wang, J. M. Humphrey, T. W. Butler, L. R. Hoth, J. B. Sperry, S. K. Sakata, C. J. Helal, C. W. am Ende, *Org. Lett.* **2018**, *20*, 812–815.

[5] R. Mamouni, M. Soukri, S. Lazar, M. Akssira, G. Guillaumet, *Tetrahedron Lett.* **2004**, *45*, 2631–2633.
